# Integrated Brain Atlas for Unbiased Mapping of Nervous System Effects Following Liraglutide Treatment

**DOI:** 10.1038/s41598-018-28496-6

**Published:** 2018-07-09

**Authors:** Casper Bo Gravesen Salinas, Tess Tsai-Hsiu Lu, Sanaz Gabery, Kasper Marstal, Tomas Alanentalo, Aaron Jeffrey Mercer, Anda Cornea, Knut Conradsen, Jacob Hecksher-Sørensen, Anders Bjorholm Dahl, Lotte Bjerre Knudsen, Anna Secher

**Affiliations:** 1 Global Research, Novo Nordisk A/S, Måløv, Denmark; 2 Image Analysis & Computer Graphics, Department of Applied Mathematics and Computer Science, Technical University of Denmark, Kgs., Lyngby, Denmark; 3 Global Research, Novo Nordisk A/S, Seattle, USA; 4 Biomedical Imaging Group Rotterdam (BIGR), Department of Radiology & Medical Informatics, Erasmus Medical Center, Rotterdam, Netherlands

## Abstract

Light Sheet Fluorescence Microscopy (LSFM) of whole organs, in particular the brain, offers a plethora of biological data imaged in 3D. This technique is however often hindered by cumbersome non-automated analysis methods. Here we describe an approach to fully automate the analysis by integrating with data from the Allen Institute of Brain Science (AIBS), to provide precise assessment of the distribution and action of peptide-based pharmaceuticals in the brain. To illustrate this approach, we examined the acute central nervous system effects of the glucagon-like peptide-1 (GLP-1) receptor agonist liraglutide. Peripherally administered liraglutide accessed the hypothalamus and brainstem, and led to activation in several brain regions of which most were intersected by projections from neurons in the lateral parabrachial nucleus. Collectively, we provide a rapid and unbiased analytical framework for LSFM data which enables quantification and exploration based on data from AIBS to support basic and translational discovery.

## Introduction

Light Sheet Fluorescence Microscopy (LSFM) is an imaging system with high sensitivity, micrometer resolution, and short acquisition time enabling imaging of a whole mouse brain in less than one hour^1^. This technology was previously used to evaluate the access of fluorescently labelled peptides from the periphery to the brain^2^. Quantification of the 3D brain distribution by manual annotation however is a slow process hindering high-throughput analysis of the large data sets. Recently, it was shown how the Common Coordinate Framework version 3 (CCFv3)^3^ of the Allen Institute for Brain Science (AIBS) can be utilized to automatically perform quantitative analysis of LSFM data based on image registration^4–6^. In the work by Reiner *et al*.^4^, the authors used the digital CCFv3 atlas as basis for quantifying whole brain activity by automated analysis of immediate early genes such as c-Fos. A similar strategy for mapping c-Fos activation using image registration was performed by Kim *et al*.^7^ and Furth *et al*.^6^. The pipeline by Furth *et al*.^6^ allows for comparison with other imaging modalities such as histology sections and neural tracing experiments by alignment of the data based on the contour of the brain. Here, we present a pipeline with a similar scope of data integration by utilizing the multi-modality nature of the AIBS atlas, which besides an average mouse brain template based on tissue auto-fluorescence^3^ also contains an aligned histology-based template constructed from Nissl-stained brain sections^8^. This enables voxel-based alignment of 3D information from LSFM together with 2D information from histological techniques, such as *in situ hybridization* (ISH), for direct data comparison. In this manuscript we utilized LSFM to map brain distribution of fluorescently labelled peptides as well as whole brain c-Fos activation. ISH was used to map the localization of the peptide’s endogenous receptor.

The AIBS data portal contains impressive amounts of open science data including expression patterns of approximately 20,000 genes in the adult mouse brain^8^, and a brain-wide, cellular-level, mesoscale connectome for the mouse describing neural connectivity between brain regions^3^. Scientists at AIBS have mapped these data to the CCFv3 atlas for efficient online browsing and quantification. However, using the online tools there is no method to automatically compare data from user experiments with content from the AIBS data portal, presenting an unmet need. Our integrated brain atlas approach allows the researcher to download data sets from the AIBS data portal and directly integrate with user LSFM experiments. Thousands of high-resolution connectivity maps of neural connections in the mouse brain from a range of mouse lines are hence available for direct numerical comparison with the user LSFM data. Together, our integrated atlas approach provides a high throughput setup allowing automated comparison and quantification of peptide brain access, receptor expression, cell activation, and brain connectivity.

The glucagon-like peptide-1 receptor agonist (GLP-1RA) was chosen as a model drug to highlight the possibilities with our analysis approach. Liraglutide is a GLP-1RA approved and widely used for the treatment of diabetes^9^, and is also approved as a treatment option for weight management, reducing appetite and body weight following peripheral once-daily administration^10^. It has been shown that GLP-1RAs act on peripheral organs but also target GLP-1 receptors (GLP-1R) in the brain. The brain actions of liraglutide are thought to contribute in large part to the weight loss effect^2,11^. Here, we first mapped the brain distribution of fluorescently labelled liraglutide following peripheral injection. The brain distribution signal was compared to the location of *glp*-*1r* expressing cells, pointing to potential peptide targets throughout the brain. Brain activation in response to peripheral administered liraglutide was quantified by mapping whole brain c-Fos activation compared to vehicle dosed controls. To increase the understanding of the activated neural networks the c-Fos findings were compared with neural connectivity maps from the AIBS data portal. Comparing the connectivity maps with liraglutide induced c-Fos increase, showed a robust correlation of activation cascades involving projections from the parabrachial nucleus (PB). Collectively, the integrated brain atlas approach combined with easy-to-use visualization tools and quantification model was shown to enable high-throughput studies aiming to investigate peptides with brain mode-of-action.

## Results

### Integrated brain atlas

**Figure 1:**
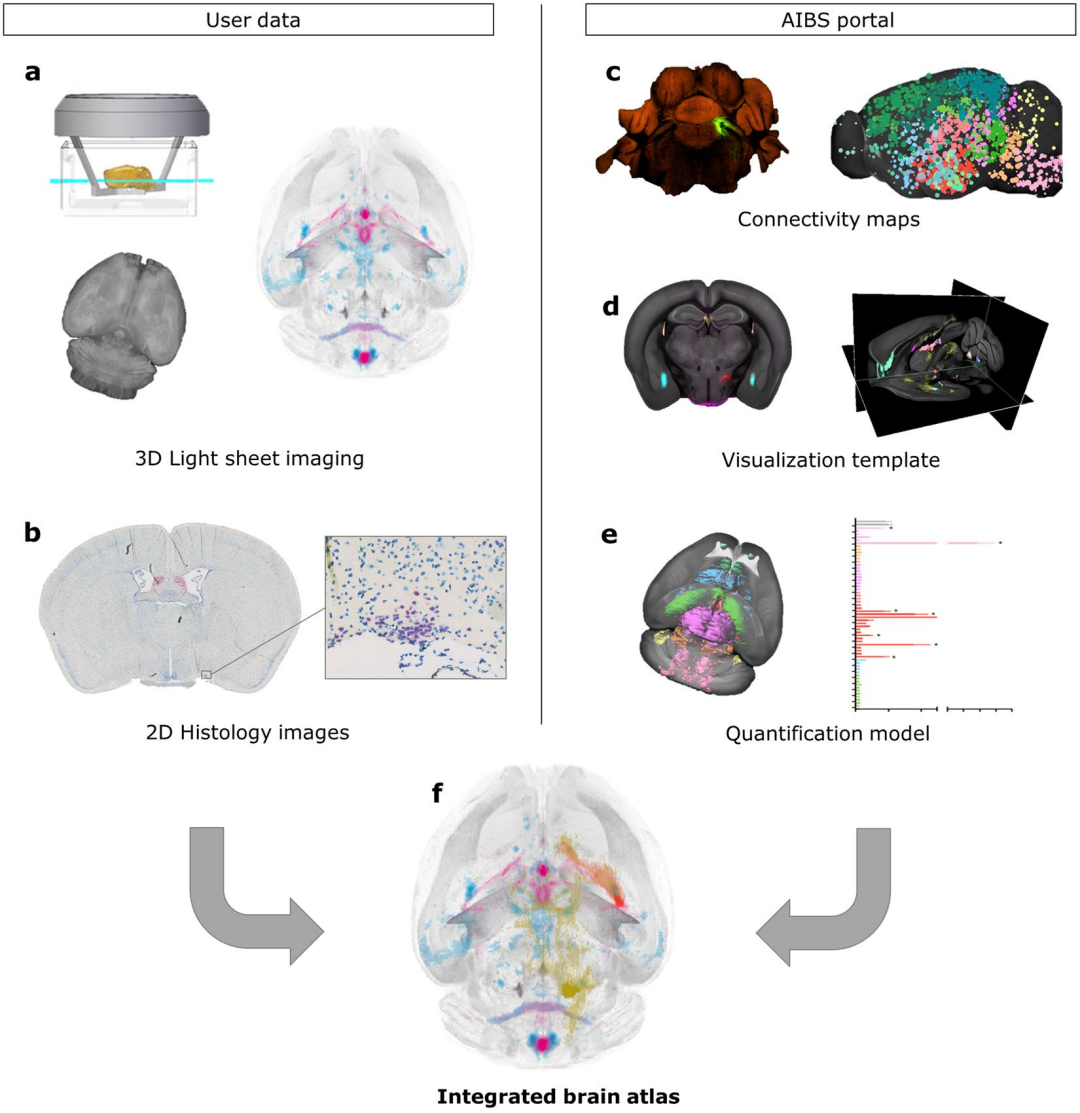
Multi-modality brain atlas integrating information from user data and AIBS data portal. (**a**) LSFM data showing brain distribution of labelled peptides (pink), as well as brain activation patterns (blue) using whole brain IHC staining for c-Fos. Top left drawing shows a mouse brain (yellow) imaged by a light sheet (green). Image credit: LaVision BioTec. (**b**) Histology data showing receptor location based on *in situ* hybridization (ISH). (**c**) Connectivity maps from AIBS^3^. Left: example of injected viral tracer. Right: Colored circles indicating the primary injection site of each experiment. © 2011 Allen Institute for Brain Science. Allen Mouse Brain Connectivity atlas. Available from: connectivity.brain-map.org. (**d**) Screen shots from a commercial 3D image rendering software used to explore overlay images of data mapped to the integrated brain atlas. (**e**) Illustration showing that all mapped data can be quantified automatically using the annotation of the CCFv3^3^. The graph data is from Fig. 2b. (**f**) Illustration of the integrated brain atlas allowing quantification and comparison between experiments from different image modalities and labelling techniques. Data can be easily navigated as maximum intensity projections (3D) or orthogonal projections (2D).

The CCFv3 from AIBS was used as basis for constructing an integrated brain atlas to enable quantification and comparison of data regarding brain access, receptor location, neural activity, and brain connectivity. The atlas implementation combines user data from LSFM and histology, as well as connectivity maps downloaded from the AIBS data portal (Fig. 1a–c). LSFM was used to image *in vivo*-dosed fluorescently labelled peptides as well as whole brain immunohistochemistry (IHC) showing c-Fos activation. Histology was used to map *glp*-*1r* expressing cells using *in situ* hybridization (ISH). Alignment to the integrated brain atlas was performed using the image registration software Elastix^12^ using affine and b-spline transformations for each of the larger brain structures (Supplementary Fig. 1). For exploration purposes, all mapped data were imported into a commercially available software program allowing visualization of 3D maximum intensity projections as well as sagittal, horizontal, and coronal 2D projection images (Fig. 1d). Visual comparisons of results from different experiments were achieved by constructing average 3D signals combining individual brain samples, and overlaying these resulting average signals onto the CCFv3 template (Supplementary Fig. 4). The average brain signals provide a compact representation of the raw data, which can be easily stored and later compared to future experiments. Automatic extraction of statistical measures in brain regions of interest was performed using the annotated model of the CCFv3 (Fig. 1e). Data presented in the following result sections were all automatically mapped to the integrated brain atlas enabling combined visualization and quantification (Fig. 1f).

### Fluorescently labelled liraglutide distributes to regions in hypothalamus and brainstem following peripheral administration

**Figure 2:**
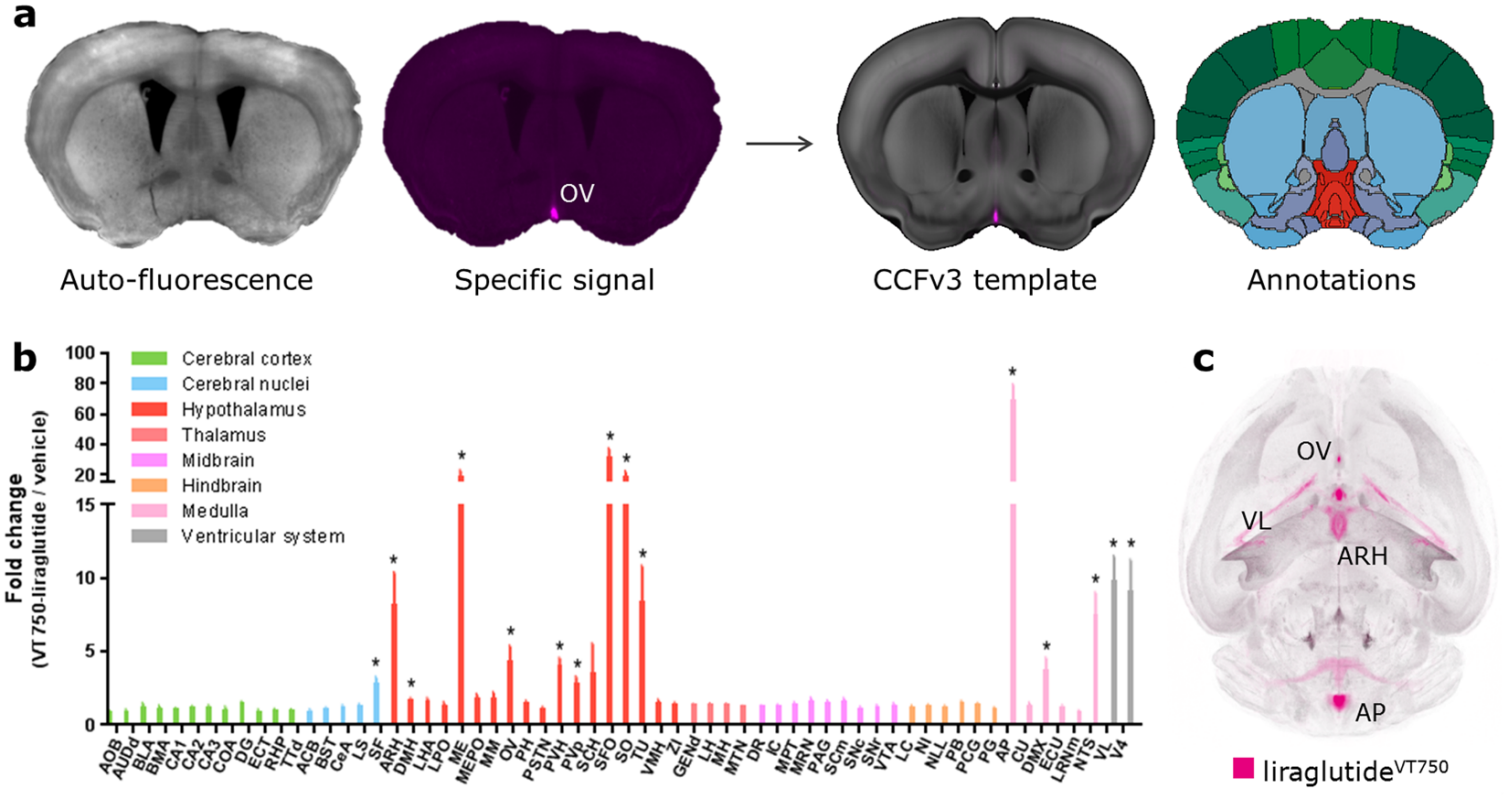
Brain regions with liraglutide^VT750^ access. (**a**) Left: 2D coronal projections from data acquired with LSFM following liraglutide^VT750^ administration in C57BL/6J mice. The specific signal was mapped to the integrated brain atlas for quantification following auto-fluorescence background reduction by spectral unmixing. Right: 2D projection showing a mapped liraglutide^VT750^ signal overlaid onto the CCFv3 atlas template^3^. Automated quantification was achieved using the corresponding annotations. (**b**) Bar graph showing the mean fold change and standard deviation (SD) of the total fluorescence signal in selected brain regions comparing liraglutide^VT750^ and vehicle (n = 5). An asterisk marks significant difference between treatments when analyzed in individual brain regions using a false discovery rate value of 5% to correct for multiple comparisons. Note the split y-axis when interpreting results and standard deviations. See Supplementary Table 1 for full brain region names. (**c**) Maximum intensity projection of the average liraglutide^VT750^ signal computed from the individual brains in the study group (n = 5).

**Figure 3:**
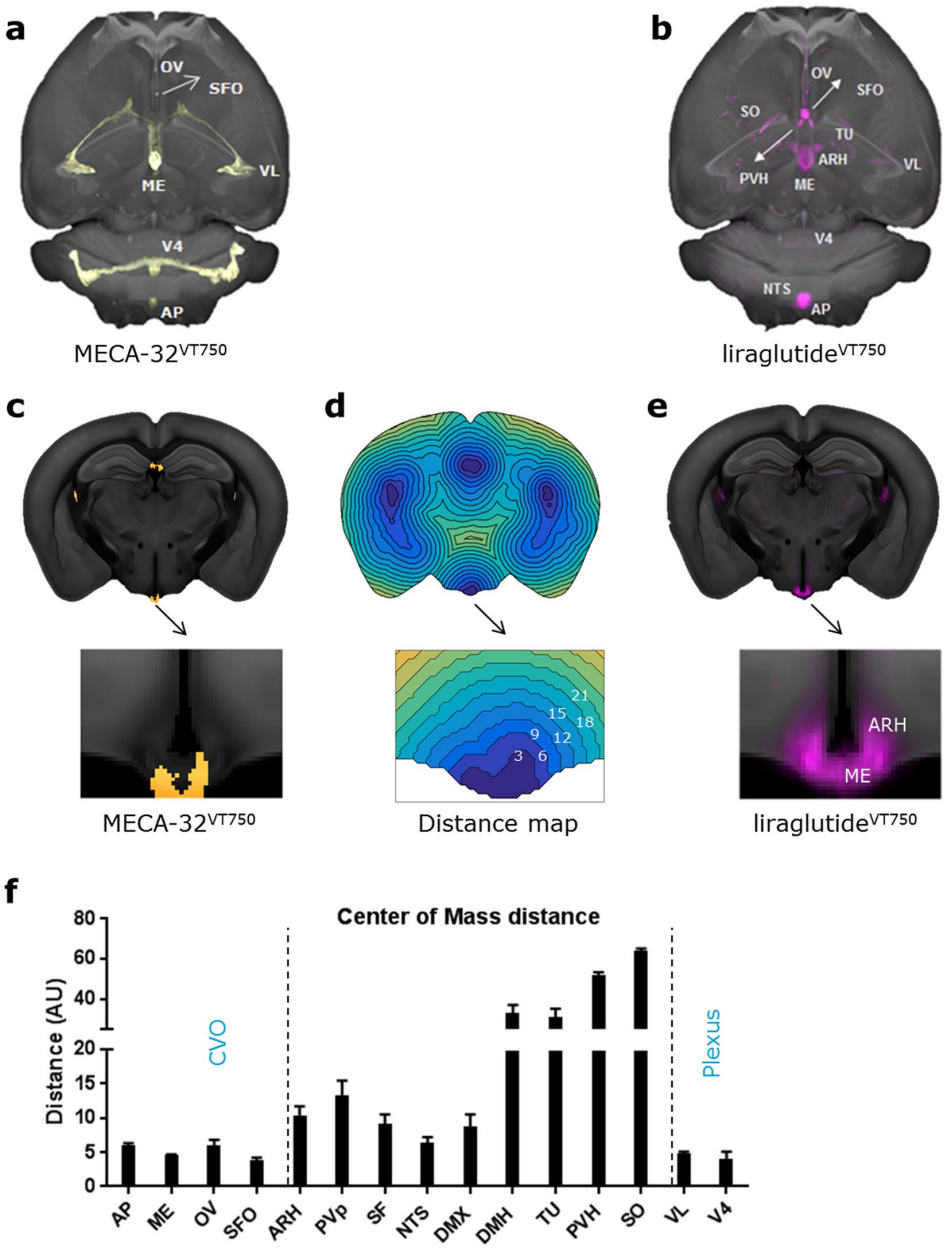
Distance map calculations. (**a**) Fenestrated capillaries visualized by maximum intensity projection of a MECA-32^VT750^ brain signal overlaid onto the CCFv3 template^3^. (**b**) Maximum intensity projection of average liraglutide^VT750^ signal (n = 5) overlaid onto the CCFv3 template^3^. (**c**) Coronal projection of the MECA-32^VT750^ signal registered to the atlas space and overlaid onto the CCFv3 template^3^. (**d**) Distance map constructed from the MECA-32^VT750^ signal. The value at a given voxel was assigned as the shortest Euclidian distance to a voxel classified as MECA-32^VT750^ positive. Blue indicate voxels close to fenestrated capillaries, while yellow indicate voxels far from fenestrated capillaries. (**e**) Average liraglutide^VT750^ signal overlaid onto a coronal section of the CCFv3 template^3^. (**f**) Center of mass distances computed for the liraglutide^VT750^ signals described in Fig. 2. The computation was performed in the brain regions marked for discovery in Fig. 2b.

GLP-1 is an incretin hormone, physiologically released from intestinal L-cell in response to food intake, but also produced and released as a neurotransmitter mainly from hindbrain neurons^13^. As the GLP-1R distribution is abundant in the brain^14,15^, we first sought to investigate which brain regions had access to the GLP-1RA liraglutide when injected peripherally. VivoTag-S®750 labelled liraglutide (liraglutide^VT750^) previously used to investigate brain access^2^ was administered intravenously in C57BL/6J mice and compared to vehicle dosed controls. The observed liraglutide^VT750^ signals were mapped to the integrated brain atlas, and the total fluorescence signal in selected regions was quantified (Fig. 2b). The analysis was enabled by registration of the auto-fluorescence channel of the LSFM acquisition to the CCFv3 template allowing quantification of the specific liraglutide^VT750^ signal using the atlas annotations (Fig. 2a). Using the nomenclature of the AIBS mouse brain atlas, liraglutide^VT750^ was significantly detected in circumventricular organs (CVOs) such as area postrema (AP) and vascular organ of the lamina terminalis (OV), as well as in regions shielded by the blood brain barrier (BBB) such as arcuate hypothalamic nucleus (ARH), paraventricular hypothalamic nucleus (PVH), and Supraoptic nucleus (SO). Liraglutide^VT750^ was also observed in the choroid plexus (chpl), exemplified by the plexus located in the fourth (V4) and lateral (VL) ventricle. To validate the findings another group of C57BL/6J mice were dosed with cy3 labelled liraglutide (liraglutide^cy3^) and the brains were processed for confocal microscopy (Supplementary Fig. 5). In general there was good agreement of targeted brain regions detected using the two different methods, except in NTS, DMX, and SF, where significantly lower signals were observed with confocal microscopy. To visualize the brain distribution of liraglutide^VT750^ an average distribution signal was computed and imported into the integrated brain atlas (Fig. 2c). It was observed that many regions with liraglutide^VT750^ access were in near proximity of the CVOs which contain fenestrated capillaries allowing free access of blood borne molecules. To elaborate on this, the distance from the CVOs to the complete liraglutide^VT750^ distribution was evaluated. This was performed by peripheral administration of fluorescently labelled MECA-32 antibody, which exclusively labels the fenestrated capillaries^16^. Figure 3 shows a maximum intensity projection of the MECA-32^VT750^ signal in the brain, together with a distance map constructed by computing the distance from any voxel in the integrated brain atlas to the nearest voxels containing MECA-32^VT750^ signal. The distance map was used to quantify the relationship between liraglutide^VT750^ and the fenestrated capillaries. The distances in most regions were small suggesting diffusion or integrated uptake of the labelled liraglutide following access through fenestrated capillaries. However, the distances in PVH, SO, and TU were significantly larger suggesting another mechanism of access to these regions.

### Aligning histology and LSFM data highlights possible direct targets of liraglutide

**Figure 4:**
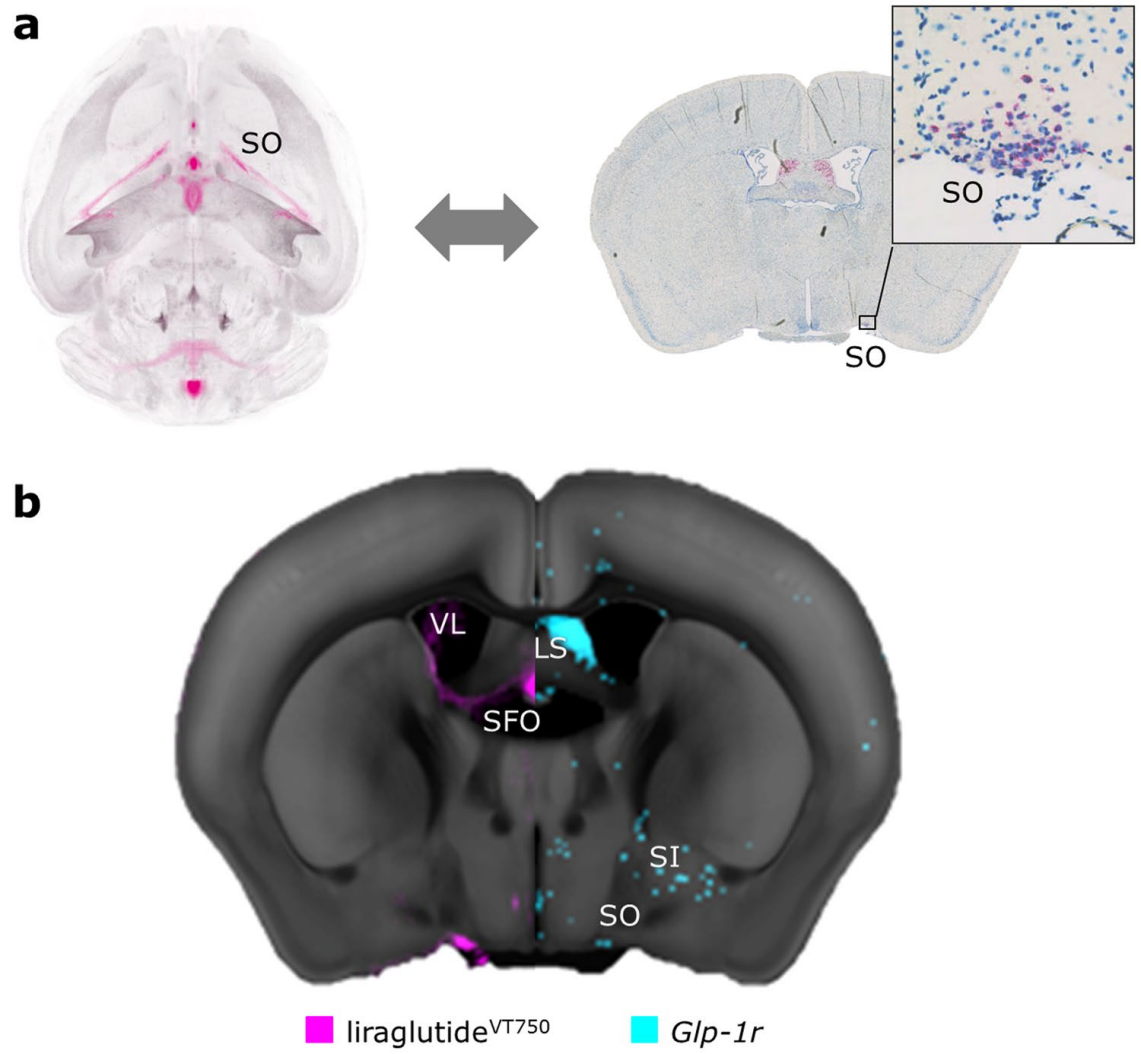
Comparison of *glp*-*1r* localization and average liraglutide^VT750^ distribution. (**a**) Concept behind aligning a 2D histology ISH image (right) with 3D LSFM data (left). A histology image was aligned to an interpolated section of the integrated brain atlas template. Once aligned a similar interpolated projection from the average liraglutide^VT750^ distribution signal could be generated. Example ISH section with zoom panel at SO region. Red color indicates cells positive for *glp*-*1r*. (**b**) Heat map (blue) representation of ISH section from (**a**) aligned with the average liraglutide^VT750^ signal (pink) from Fig. 2c. The signals are overlaid onto separate halves of the CCFv3 template^3^.

To have a direct brain effect the administered liraglutide must activate its receptor, the GLP-1R. To further elaborate the relevance of the brain distribution of liraglutide^VT750^ a comparison was made between the average distribution signal and cells expressing *glp*-*1r* mRNA. The ISH platform RNAscope^17,18^ was used to map the *glp*-*1r* mRNA location in a number of coronal slides throughout the C57/BL6 mouse brain. To enable direct comparisons of images from 2D histology with 3D LSFM imaging (Fig. 4a), a heat map was computed from the ISH section to represent the *glp*-*1r* positive cells. The heat map was aligned to the integrated brain atlas, which enabled direct visual comparison with the average liraglutide^VT750^ distribution from Fig. 2c. An example of a mapped *glp*-*1r* ISH heat map compared to the corresponding anatomical location in the average liraglutide^VT750^ distribution signal is seen in Fig. 4b. In this anatomical location the liraglutide^VT750^ distribution signal overlapped with the *glp*-*1r* signal in SO and SFO, while a liraglutide^VT750^ signal was also observed in the chpl located in VL where no receptor signal was present. Receptor expression was observed in Lateral septal nucleus (LS), Substantia innominata (SI) and the anterior part of PVH without a corresponding liraglutide^VT750^ signal. More *glp*-*1r* ISH images were obtained for regions with liraglutide^VT750^ access (Supplementary Fig. 6). Comparing to the statistical significant brain regions with liraglutide^VT750^ access (Fig. 2b), *glp*-*1r* was detected in ARH, DMH, PVH, OV, PVp, SO, TU, AP, and NTS providing possible direct targets of liraglutide. To further investigate liraglutide^VT750^ interaction with the GLP-1R a group of *Glp*-*1r*^−/−^ mice was studied. In parallel to the liraglutide^VT750^ distribution study presented in the previous section, liraglutide^VT750^ was injected in *Glp*-*1r*^−/−^ mice devoid of a functional GLP-1R. Visual and quantitative comparisons of the distribution signals showed a large decrease of signal in the knockout mice, in all brain regions except for the choroid plexus (Supplementary Fig. 7). Together with the histology findings, this indicated a specific GLP-1R dependent localization of liraglutide^VT750^ in the brain.

### Whole brain c-Fos staining shows that liraglutide activates brain regions related to food intake

**Figure 5:**
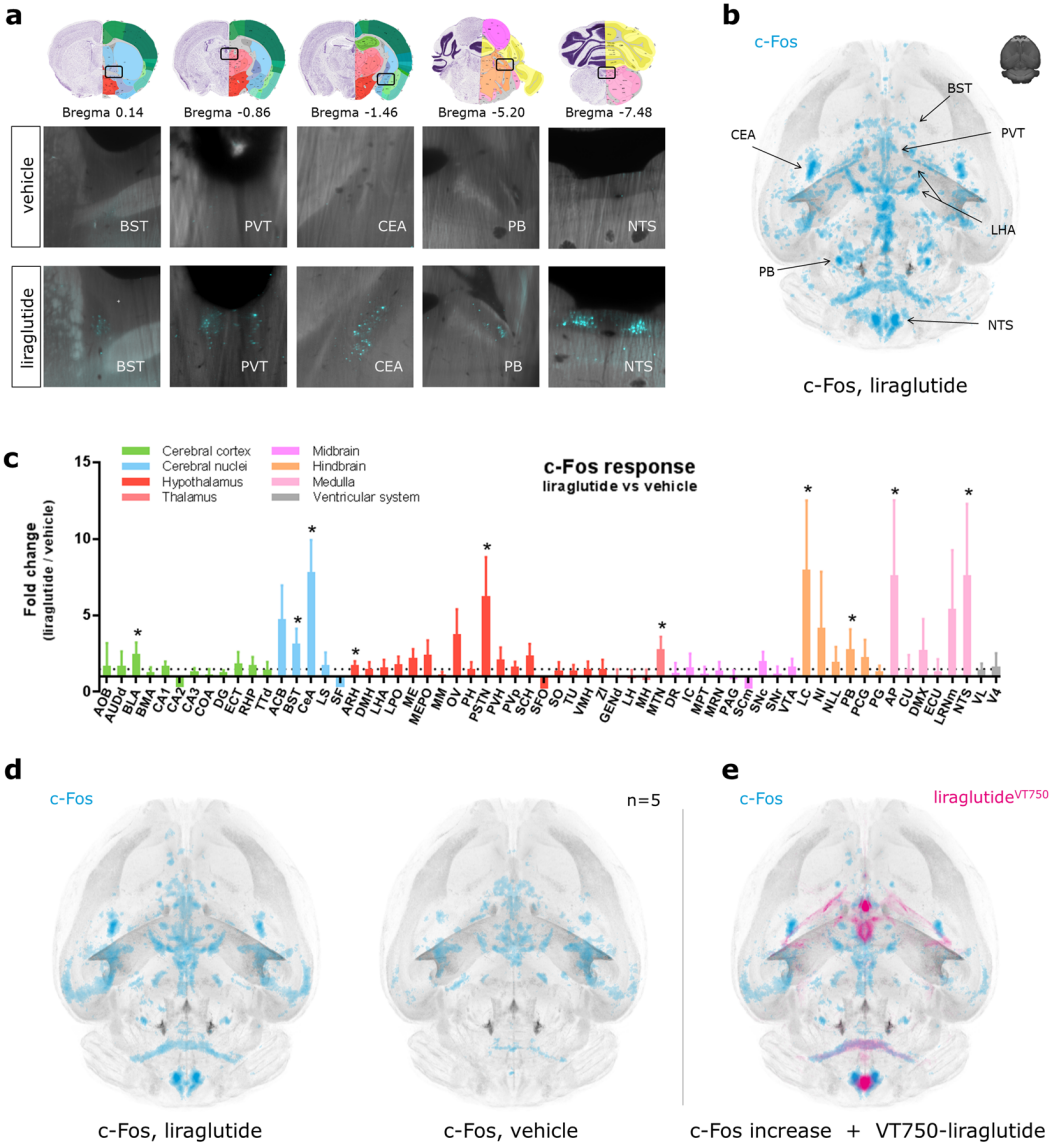
Neural activation following liraglutide administration. (**a**) Example images of c-Fos response in liraglutide vs vehicle dosed C57BL/6J mice. Bregma coordinates denote the anatomical location together with coronal atlas plates. Image credit: Allen Institute. (**b**) c-Fos heat map computed from one liraglutide injected mouse. See also Supplementary Fig. 8. (**c**) Bar graph showing the mean fold change and standard deviation (SD) of total c-Fos heat map signal in selected brain regions comparing liraglutide and vehicle dosed animals. Regions were selected as having either liraglutide^VT750^ access, GLP-1R expression, or c-Fos response. An asterisk marks significant difference between treatments when analyzed in individual brain regions using a false discovery rate value of 20% to correct for multiple comparisons. See Supplementary Table 1 for full brain region names. (**d**) Computed average c-Fos signal from liraglutide injected animals (left), and vehicle injected animals (right). (**e**) Liraglutide specific c-Fos increase overlaid with the average liraglutide^VT750^ distribution from Fig. 2c.

GLP-1RAs are known to reduce appetite following peripheral administration, and this effect has been linked to both hypothalamic and hindbrain action^2,19^. To investigate changes in brain activity following peripheral administration of liraglutide, C57BL/6J mice were dosed subcutaneously with liraglutide, and the c-Fos response was compared to vehicle dosed controls. c-Fos staining was performed on intact brains using the iDISCO protocol^20^, and brains were subsequently scanned using LSFM. The most prominent c-Fos increase following liraglutide injection was seen in Bed nuclei of the stria terminalis (BST), Paraventricular nucleus of the thalamus (PVT), Central amygdala nucleus (CEA), Parabrachial nucleus (PB), and NTS (Fig. 5a). To evaluate the whole brain c-Fos response, heat maps were computed for each individual brain sample based on the amount of c-Fos positive cells, and these heat maps were registered to the integrated brain atlas (Fig. 5b). The heat maps were used to quantify the differences between liraglutide and vehicle dosed animals in select brain regions (Fig. 5c). To investigate whether the observed c-Fos response could be a direct effect of liraglutide interaction with GLP-1R we visually compared the liraglutide specific c-Fos increase with the liraglutide^VT750^ distribution from Fig. 2c. This was enabled by first computing the average c-Fos response from the liraglutide and vehicle group separately (Fig. 5d), followed by voxel wise subtraction of the vehicle response from the liraglutide response. The liraglutide specific c-Fos increase was then imported into the integrated brain atlas to allow direct overlay with the average liraglutide^VT750^ distribution signal (Fig. 5e and Supplementary Movie 1). Some overlap was seen, mainly in AP, but also in ARH and OV indicating possible direct activation from liraglutide in these regions. However, the majority of c-Fos increase occurred in regions not directly targeted by liraglutide^VT750^, indicating secondary activation. Supplementary Fig. 9 shows a rank plot comparing the ranks from lowest to highest fold change of data presented in Figs 2b and 5c to give a further overview of brain regions with possible direct and secondary activation. c-Fos increase but no liraglutide^VT750^ signal, indicating secondary activation, was seen in BST, PVT, PB, and CeA which are regions previously described to be related to lowering of food intake^19,21^. Liraglutide specific c-Fos increase was also observed in the motor neurons receiving input from the vagus nerve.

### Comparing c-Fos increase with AIBS connectivity maps suggests that activation may involve a common parabrachial pathway

**Figure 6:**
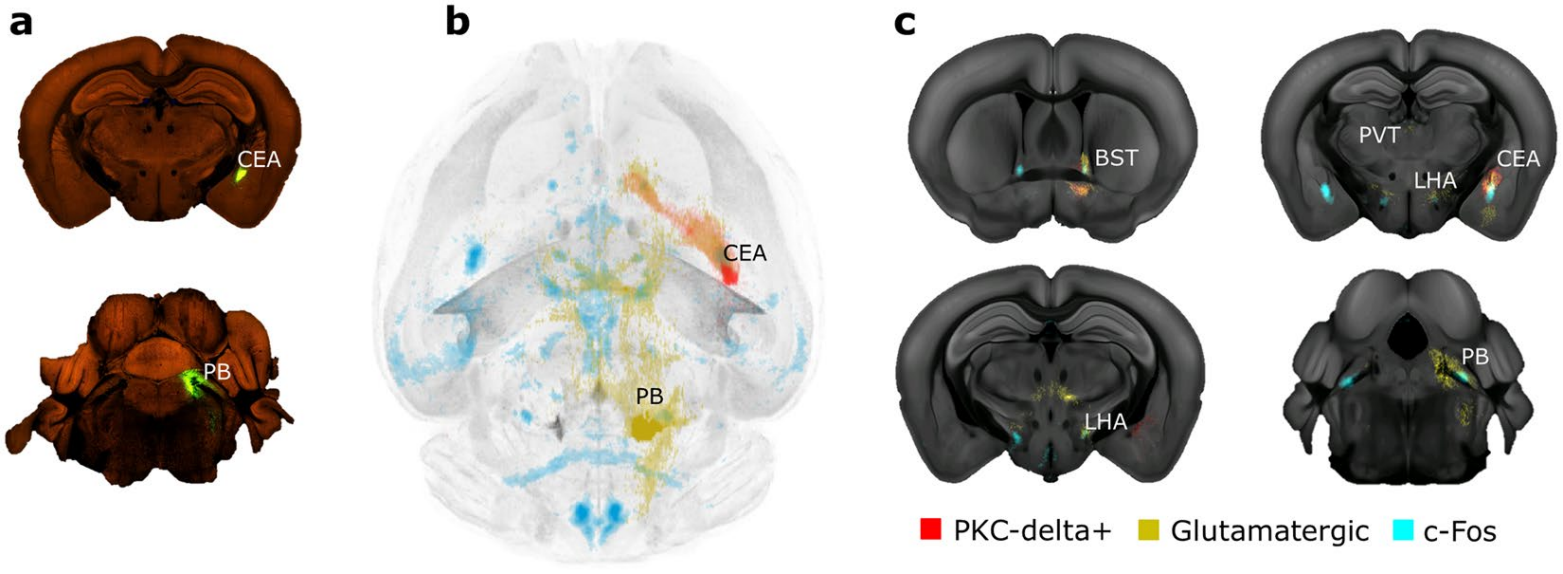
Comparing brain connectivity maps with whole brain c-Fos data. (**a**) Top: Part of primary injection site (CEA) in a Prkcd-GluCla-CFP-IRES-Cre mouse. Bottom: Part of primary injection site (PB) in a slc17ac cre mouse. ©2011 Allen Institute for Brain Science. Allen Mouse Brain Connectivity atlas. Available from: connectivity.brain-map.org. (**b**) Connectivity maps visualized by horizontal maximum intensity projection overlaid with the average c-Fos increase following liraglutide administration from Fig. 5e. (**c**) Coronal projection images showing downloaded glutamatergic projections from PB, and PKD-delta+ projections from CEA, together with the average c-Fos increase following liraglutide administration. Signals are overlaid onto the CCFv3 template^3^.

The fact that the majority of induced brain activity seen by c-Fos increase was observed in brain regions not directly targeted by liraglutide^VT750^ likely indicates that neurons in these regions responded as part of larger neural networks. We hypothesized that these regions were part of networks related to appetite regulation and feeding behaviour. To investigate this hypothesis the complete set of grey matter connectivity maps were downloaded from the AIBS data portal (2469 experiments) and mapped together with the liraglutide specific c-Fos increase. The 2469 connectivity maps were automatically ranked by the amount of overlap with the c-Fos increase, taking into account the total overlap of the c-Fos signal and given connectivity signal in relation to the amount of c-Fos signal not overlapping with the given connectivity signal and vice versa (Fig. 6). The top 30 ranking results of the c-Fos to connectivity maps comparisons can be viewed in Supplementary Table 2. Based on the automated ranking, the most overlapping connectivity map was projections from Rasgrf2 (Rasgrf2-2A-dCre) positive cells in the NTS. In addition, several neuronal subpopulations previously described to decrease food intake were observed among the highest ranking connectivity maps. Projections from proopiomelanocortin (POMC) positive neurons (Pomc-Cre (BL)) in the ARH were ranked as the 29^th^ most overlapping connectivity map, and targeting of this neuronal subpopulation has previously been shown following GLP-1RA stimulation^2^. Projections from PKC-delta+ positive neurons in CEA were ranked as the 15th most overlapping connectivity map. PKC-delta+ is a neuronal subpopulation which has previously been described to influence anorexigenic signals in combination with the lateral PB^22^. Based on the automated ranking, projections from the lateral PB were seen in four of the ten most overlapping connectivity maps compared to the liraglutide specific c-Fos increase. As liraglutide^VT750^ showed no significant access in the PB, this suggests a role of the PB as a relay station of input from other targeted brain regions. Glutamatergic (slc17a6-IRES-Cre) projections from the lateral PB (4^th^ most overlapping connectivity map) and PKC-delta+ (Prkcd-GluCla-CFP-IRES-Cre) projections from the CEA were imported into the integrated brain atlas to visualize how these neurons could be part of the activated c-Fos response following liraglutide treatment (Fig. 6 and Supplementary Movie 2). This parabrachial pathway has in recent literature been described as a general circuit causing anorexia^19,22,23^, and here also appears to be involved in the acute brain activation of liraglutide.

## Discussion

In this manuscript an easy to use analysis approach was presented, combining data from numerous sources in an integrated brain atlas including data from LSFM imaging, histology, and data from the AIBS. LSFM imaging is high throughput with data acquisition times in the range of one hour for a whole mouse brain. The automated analysis approach enables the researcher to gain the advantages of this high throughput LSFM imaging system by removing the bottleneck arising from manual analysis of the large data sets. By registering the acquired whole brain data with the CCFv3 atlas from AIBS, the user can additionally align LSFM data with standard histological images, as well as automatically compare LSFM data to connectivity maps from the AIBS data portal. By combining the different imaging technologies the distribution of bioactive peptides can be correlated to the peptide’s endogenous receptor, changes in brain activity, and connectivity between relevant brain regions. This provides mechanistic information that would not be afforded by a single technology alone. Furthermore, importing all experiment data to a common reference atlas effectively allows the user to build an anatomical database of information which can be compared to parallel and future studies, thereby increasing the value of each experiment. It should be noted that the brain atlas is based on adult C57BL/6J mice with normal brains. The analysis will not be as accurate in animal models with significant neuron loss, as the atlas and brain sample would not contain the same information.

To highlight the possibilities with this analysis approach the acute brain effect of the GLP-1RA liraglutide was investigated. In agreement with previous observations^2^, liraglutide^VT750^ had access to the chpl, the CVOs, and regions shielded by the BBB such as ARH, PVH, SO, and TU. It should be noted that the observed liraglutide^VT750^ signals in regions close to the CVOs may have been overestimated due to the thickness and beam profile of the light sheet in the LSFM system. This was the case for the brain regions NTS, DMX, and SF comparing to detection with confocal microscopy. To minimize this issue work is needed concerning deconvolution algorithms for LSFM systems. The issue was much less pronounced in the c-Fos studies as the fluorescence signals here were much more uniform and confined to the cell bodies. By computing the distance between the fenestrated capillaries and the average liraglutide^VT750^ distribution, it was seen that the peptide likely could access the brain either by diffusion from the CVOs or by uptake through specialized cell structures intercepting the BBB protected brain regions with the CVOs. Examples of such cells are tanycytes which have previously been demonstrated to mediate uptake of other peripheral peptides in the ARH^24^. *Glp*-*1r* expression was observed in most brain regions with liraglutide^VT750^ access indicating that the observed liraglutide^VT750^ distribution signal was GLP-1R dependent. This was confirmed by a decreased brain distribution signal of liraglutide^VT750^ observed in *Glp*-*1r*^−/−^ mice. Previous experiments with liraglutide^VT750^ in wild type and *Glp-1r*^−/−^ mice showed a marked difference in uptake with only choroid plexus uptake in *GLP-1r*^−/−^ mice, leading to the conclusion that the uptake is GLP-1R dependent uptake^2^. Changes in brain activation following an acute subcutaneous injection of liraglutide were investigated by whole brain c-Fos staining. Visually the most prominent increases were seen in BST, PVT, CEA, PB, and NTS, while quantitative analysis also showed a significant increase in AP and ARH. Comparing the c-Fos increase to the liraglutide^VT750^ distribution it was possible that liraglutide directly activated GLP-1R in AP and ARH. c-Fos signal could not be observed in some regions with strong liraglutide^VT750^ signal such as SO, TU and SFO. Although not demonstrated before, this could be due to time related differences in protein activation of primary versus secondary activation sites or negative activation profiles counteracting the signal. As c-Fos is a marker for increased neuronal activity it is also possible that some of the effect of liraglutide is inhibitory, and hence not detected by the c-Fos analysis. It should also be noted that c-Fos response to a stimuli such as a pharmaceutical agent may depend on the study setup. Activity status will not be evident by c-Fos in all neuronal populations and lack of signals will only be detected following prior challenge with other stimuli. Furthermore, variations could be related to circadian rhythm, fasted vs fed state etc. In the present study animals were fed ad lib and dosed 4 hours after lights on. A comparison between the liraglutide^VT750^ distribution signal and the liraglutide specific c-Fos increase showed that the majority of brain activation was likely to be secondary events. To understand the secondary c-Fos activation 2469 connectivity maps from the AIBS data portal were downloaded and added to the integrated brain atlas. Projections from ras-specific guanine-nucleotide releasing factor 2 (Rasgfr2) positive cells in NTS were ranked by automated computations as the most overlapping connectivity map comparing to the liraglutide specific c-Fos increase. Previous work indicates a role for Rasgfr2 in the regulation of mesolimbic dopamine neuron activity^25^. However, to the best of our knowledge no direct links has been established with appetite or body weight regulation. A strong correlation between c-Fos activation and connectivity maps were also seen with projections from the lateral PB exemplified by glutamatergic projections (slc17a6 cre). Recent work^19,23,26,27^ describes the role of the lateral PB as a conduit for visceral signals that cause anorexia. The lateral PB has also been shown to be necessary for the acute reduction in food intake by another GLP-1RA exendin-4 in rats^28^. The lateral PB is a site of integration of satiety signals from the brainstem, but other neurons also projects to this region such as agouti-related protein (AgRP) and POMC positive neurons from the ARH. This suggests an interconnection between brainstem and hypothalamus input in the control of feeding. Direct projections from POMC positive neurons in the ARH also showed a general overlap with the liraglutide specific c-Fos increase, although to a lesser degree compared to projections from the lateral PB.

As all experiments were registered to a common atlas space the quality of subsequent data analysis was dependent on the registration quality. The 3DISCO clearing protocol^29^ was used prior to LSFM imaging which was previously reported to introduce non-uniform tissue shrinkage^4^. To mitigate the effects of the non-uniform tissue shrinkage a registration model which allowed different degrees of shrinkage in each of the larger brain structures was introduced. The registration accuracy was tested by manually placing landmarks in the atlas template and comparing these to corresponding landmarks placed in a test set of registered brain samples. The average error between corresponding landmarks was approximately 5 voxels equal to 100 μm (Supplementary Fig. 2). The LSFM system acquires high resolution images from which lower level representations such as average distribution signals and c-Fos heat maps of brain activity were computed. These representations enabled direct visual and quantitative comparisons of results from parallel experiments. The strategy of automated quantification based on brain atlases is becoming well established in the field. For example, ClearMap^4^ and WholeBrain^6^ are two recent publicly available data processing pipelines. Both pipelines robustly maps brain activity using c-Fos and integrate with AIBS. ClearMap is used through Python, while WholeBrain is used through R. Our pipeline can be used through the graphical user interfaces of Imaris and Excel and thus requires no scripting by the user. Overall, the developed analysis approach provides a strong foundation for anatomical studies and could be a valuable tool in combination with functional studies going forward. The high throughput approach enables automated analysis of user experiments based on large scale data from AIBS, and by integrating these data the goal is to discover new knowledge otherwise hidden in the large complexity of the brain.

## Methods

### Animals

All *in vivo* studies were conducted in accordance with approved national regulations in Denmark, which are fully compliant with internationally accepted principles for the care and use of laboratory animals, and with animal experimental licenses granted by the Danish Ministry of Justice. Animals were obtained from Taconic, Denmark and housed in standard, temperature-controlled conditions with a 12-hour-light/dark cycle. The animals had ad libitum access to water and regular chow.

### Distribution of liraglutide^VT750^

Fluorescently labelled liraglutide was synthesized by conjugating VivoTag-S®750 NIR FLUOROCHROME LABEL (Perkin Elmer) to the liraglutide peptide. Liraglutide^VT750^ (30 nmol/kg) or vehicle (PBS) were injected I.V. into male (n = 6) C57BL/6J wild type and *Glp*-*1r*^−/−^ mice, and left to circulate for 6 hours in the animals.

### Distribution of MECA-32^VT750^

Fluorescently labelled MECA-32 antibody was synthesized by conjugating VivoTag-S®750 NIR FLUOROCHROME LABEL (Perkin Elmer) to the MECA-32 antibody (BE0200) from BioXCell. MECA-32^VT750^ (0.2 mg/kg) were injected I.V. into male (n = 2) C57BL/6J wild type mice, and left to circulate for 6 hours in the animals.

### Distribution of liraglutide^Cy3^

Fluorescently labelled liraglutide was synthesized by conjugating Cyanine 3 (Cy3) (Lumiprobe) to the liraglutide peptide. Liraglutide^Cy3^ (120 nmol/kg) or vehicle (PBS) were injected I.V. into male (n = 3) C57BL/6J mice and left to circulate for 6 hours in the animals.

### c-Fos activation after liraglutide

Liraglutide (0.4 mg/kg) or vehicle (pH = 8.15; 50 nM phosphate; 70 nM sodium chloride; 0.05% polysorbate 80) were injected S.C into male (n = 6) C57Bl/6J mice and left to circulate for 4 hours in the animals.

### Sample preparation

Mice were euthanized using isofluorane followed by cardiac perfusion with 10% neutral buffered formalin (NBF) (liraglutide^VT750^ and MECA-32^VT750^) or 4% paraformaldehyde (PFA) (liraglutide^Cy3^, liraglutide), followed by fixation in NBF (liraglutide^VT750^ and MECA-32^VT750^) or 4% PFA (liraglutide^Cy3^, liraglutide) at room temperature overnight before further processing.

### Tissue sectioning and staining

Brain samples for evaluation of liraglutide^Cy3^ distribution were sectioned. After post fixation, the brains were transferred to 25% sucrose solution. Once the brains had descended to the bottom of the well, they were cryosectioned coronally at 35 µm and collected in 6 series. One series from each mouse was stained with DAPI (1:1000).

### Whole brain IHC

Brain samples were transferred stepwise to 100% methanol (MeOH) using the following protocol: 20% MeOH in demineralised H_2_O (dH2O) for 1 hour, 40% MeOH for 1 hour, 60% MeOH for 1 hour, 80% MeOH for 1 hour, and 2 × 100% MeOH for 1 hour each. iDISCO staining was performed as described in Renier *et al*.^20^ with one exception: All the washing steps after incubation with primary and secondary antibody were extended from one to three days. Incubation times for primary and secondary antibody were 4 days each. The primary antibody was Rabbit pAb anti c-Fos (Ab-5) (4–17) from Calbiochem (1:10,000 dilution), and the secondary antibody was Cy5-anti rabbit from Jackson Immunoresearch (1:1000 dilution).

### Tissue clearing

Liraglutide^VT750^ and MECA-32^VT750^ brain samples were cleared using the 3DISCO protocol^29^. In brief, brain tissue was dehydrated at room temperature in graded tetrahydrofuran (THF) diluted in dH2O (w/v) 50/80/96/99%/2 × 100% 6–12 hours for each step. The brains were then cleared at room temperature in 3 × diBenzylether (DBE) for 6–12 hours. Note that for future work, the authors recommend using the clearing protocol associated with iDISCO+ instead^4^. This protocol has been validated for clearing of liraglutide^VT750^ brain samples and was used to clear the brains in the c-Fos activation study. In brief, brain tissue is dehydrated at room temperature in graded MeOH diluted in dH_2_O (w/v) 20/40/60/80%/100% for 1 hour/1 hour/1 hour/1 hour/24 hours respectively. Tissue is then cleared by a mix of 66% Dichloromethane (DCM) with 33% MeOH for 3 hours followed by 100% DCM twice for 15 minutes, followed by 2X DBE for 24 hours.

### Light sheet imaging

Liraglutide^VT750^, MECA-32^VT750^, and c-Fos brain samples were imaged by LSFM. The scan parameters were chosen to balance data acquisition time and resolution. Image stacks (16 bit tiff) were acquired using an UltraMicroscope II LSFM system (Lavision Biotec, Bielefeld, Germany) equipped with an Andor Neo 5.5 scmos camera (Andor Technology Ltd., Belfast, UK) in 10.32 μm isotropic resolution for the liraglutide^VT750^, and MECA-32^VT750^ distribution study, and 4.06 μm for the c-Fos activation study. Data acquisition was performed using a 620/60 nm excitation filter and 680/30 nm emission filter (liraglutide^VT750^, MECA-32^VT750^) or 545/30 nm excitation filter and 620/60 nm emission filter (c-Fos) for imaging auto-fluorescence, and a 710/75 nm excitation filter and a 780/40 nm emission filter (liraglutide^VT750^, MECA-32^VT750^) or a 620/60 nm excitation filter and 680/30 nm emission filter (c-Fos) for imaging specific signals.

### Confocal imaging

Liraglutide^Cy3^ distribution was visualized by confocal imaging. Images from ARH, OV, PVH, PVp, SFO SO TU and AP/NTS were acquired with the Leica Confocal Imaging System TCS SP8 with the HC PL APO 20x/0.75 objective. Confocal Z-stacks were acquired ranging between 12–25 µm with laser excitation in sequential mode, corresponding to DAPI and Texas red, from all the regions. The Z-stacks were then processed into maximum projection images using the Leica Application Suite (LAS X) software.

### RNAscope ISH

C57BL/6J male mice (n = 2) were perfused and post-fixed as described previously^30^ and tissues were subsequently paraffin-embedded and sectioned at 5 μm intervals around brain regions marked for discovery in Fig. 2b. Automated ISH was performed on a Ventana Discovery ULTRA following the technical bulletin provided by ACD Bio (#322250-USM-ULT). Our assay utilized the Ventana mRNA RED Detection Kit (Roche, #7074654001), RNAscope RED kit (ACD Bio, #322250), and the following oligonucleotide probes from ACD Bio: Mm-Glp1r (#418859), Mm-Polr2a (#312479), Mm-NPY (#313329), and dapB (#312039). Mm-Polr2a and Mm-NPY served as positive controls, and dapB was used as a negative control. ISH sections were subsequently counterstained in 30 dips of Mayer’s hematoxylin, coverslipped, and scanned on a Zeiss AxioScan at 40× magnification. To assist in aligning ISH data with the integrated brain atlas, Nissl-staining (0.1% w/v cresyl violet with 0.5% v/v glacial acetic acid, 5 minute incubation) was performed on adjacent brain sections from each series of tissue collected for ISH.

### Brain atlas

The CCFv3^3^ greyscale anatomical atlas template and corresponding annotation volume were downloaded in 25 µm isotropic resolution from AIBS. Additionally, the greyscale Nissl-based atlas template from the Allen Mouse Brain Atlas was downloaded. The outer part of the olfactory bulb was cropped away and zero padding were added in all dimensions. The AIBS atlas ontology was downloaded and used to construct a custom annotation volume corresponding of 564 individual brain regions. A representative mouse brain acquired with LSFM was spatially aligned to the modified CCFv3 anatomical atlas template using image registration. All brain atlas components were saved as nifti files and made available at http://BrainPatterns.compute.dtu.dk together with a list of included brain regions.

### Image registration

3D LSFM images and 2D slide scanner images were registered using the Elastix software library^12^. The source image *I*_*S*_(*x*) was registered to the target image *I*_*T*_(*x*) by finding a coordinate transformation *T*(*x*) that made *I*_*S*_(*T*(*x*)) spatially aligned with *I*_*T*_(*x*). We used an affine coordinate transformation for initialization followed by non-rigid b-spline coordinate transformation. The alignment was iteratively optimized with respect to the mutual information between the source and the target^12^. Registration parameters for mapping of LSFM data were similar to those used in Renier *et al*.^4^. Parameters for registration of histology sections were similar to those used in Abdelmoula *et al*.^31^. All parameter files are available at: http://BrainPatterns.compute.dtu.dk.

### Quantification of brain distribution

Spectral unmixing was performed to minimize the contribution of tissue auto-fluorescence in the liraglutide^VT750^ distribution study. The estimated auto-fluorescence contribution in the specific channel was calculated and removed based on ratios of voxel intensities between selected voxels in the unspecific channel and the corresponding voxels in the specific channel. A ratio was computed for 40 sets of voxels selected from the histogram of the unspecific channel. An example of applying the unmix algorithm is seen in Supplementary Fig. 3. Quantification was performed by summation of the unmixed intensity value of all voxels within the individual brain regions. The results were reported as fold change between the study groups.

### Distance map computations

The MECA-32^VT750^ and average liraglutide^VT750^ distribution signals were aligned to the atlas space by image registration and binarized by thresholding. A distance map was constructed by assigning the value at a given voxel as the shortest Euclidian distance to a voxel classified as MECA-32^VT750^ positive. The center of mass distances presented in Fig. 3f were calculated brain region-wise based on the liraglutide^VT750^ intensities and the distance map.

### Quantification of brain activation

A cell segmentation algorithm was used prior to construction of c-Fos brain activation heat maps. The cell segmentation algorithm was applied to each acquired 2D image of the LSFM scan sequence one by one. Cells are blob-like features, and therefore a blob-detection filter was applied based on computing two eigenvalues of the hessian matrix in each pixel in the image of the specific channel. A feature was constructed by addition of the eigenvalues to enhance the contrast of blob shaped objects in the image. Top-hat filtering was used to remove uneven background illumination from the image while emphasizing spherical shaped objects^32^. For six iterations the sequence of blob filtering and top-hat filtering was repeated to separate overlapping clusters of cells into individual cells. Finally, a binary image of the pixels containing positive stained cells was constructed by thresholding. After cell segmentation, a heat map was created by summing uniform intensity discs with diameter of 20 μm placed on each pixel representing a positive stained cell. The heat maps were later used for visualization and quantification. Quantification was performed by summation of the heat map intensity value of all voxels within the individual brain regions, indicating the total brain activation signal in these regions. The results were reported as fold change between the study groups. As the algorithm operates in 2D the user should note that cells may contribute to the heat map more than once if the physical distance between LSFM scan planes is less than the diameter of the cells.

### Connectivity map ranking

The available connectivity maps were downloaded from the AIBS data portal. The connectivity maps were ranked according to the voxel-wise overlap with the liraglutide specific c-Fos increase presented in Fig. 5e. The ranking was performed using the following measures of overlap between the c-Fos signal and the individual connectivity signals: (1) Overlap computed as the sum of voxels containing both c-Fos increase signal and connectivity signal divided by the sum of voxels containing either c-Fos increase signal or connectivity signal. This number measures the overlap of signals to the union of the signals. (2) Overlap computed as the sum of voxels containing both c-Fos increase signal and connectivity signal divided by the sum of voxels containing connectivity signal. This number measures the overlap relative to the connectivity signals alone. (3) Overlap computed as the sum of voxel intensities from voxels containing both c-Fos increase signal and connectivity signal divided by the sum of voxel intensities of voxels containing c-Fos increase signal. This number measures the overlap relative to the c-Fos signal alone. Note that the third overlap measure uses intensities, i.e. emphasizes regions with larger c-Fos increase. The final ranking was performed as an equally weighted average of the rankings based on the three overlap measures.

### Statistical analysis

ROI based analysis of the total brain distribution signal intensities presented in Fig. 2b, and c-Fos heat map intensities in Fig. 4c, were computed using multiple t-tests assuming unequal variance. Statistical significance was determined by correcting for multiple comparisons using a false discovery rate set to 5% for labelled peptides and 20% for c-Fos heat maps. The statistical analysis was performed using Graphpad Prism (Release 6, GraphPad Software, Inc., United States). Statistical results of multiple t-tests on all brain regions are included in Supplementary Tables 3 and 4. Ranking of connectivity maps were performed as described above.

### Matlab executables

All image analysis algorithms were written in Matlab (Release 2012b, MathWorks, Natick, Massachusetts, United States) with image registration done through calls to Elastix^12^. The Matlab scripts are compiled as executables and provided as ImarisXT plugins to use directly from within Imaris (Release 7.6.5, Bitplane, Zurich, Switzerland). The atlas files are available as nifti files, and can be imported in Imaris for 3D visualization using the provided plugins. Imaris and Matlab were used to generate all figures in the manuscript, except Supplementary Fig. 1e which was generated using SimpleElastix^33^_._ Files, installation guide, and user guide are available for download at http://BrainPatterns.compute.dtu.dk.

### Data availability

The datasets generated during the current study, if not already available on http://BrainPatterns.compute.dtu.dk, are available from the corresponding author on reasonable request. Available software is licensed with a GPLv3 license.

## Electronic supplementary material

Supplementary Information

Comparing liraglutideVT750 brain distribution with liraglutide specific c-Fos increase

Comparing liraglutide specific c-Fos increase with AIBS connectivity maps
