# Supplementary material for: Integrated Brain Atlas for Unbiased Mapping of Nervous System Effects Following Liraglutide Treatment
Source: Sci Rep. 2018 Jul 9;8:10310. doi: 10.1038/s41598-018-28496-6 (PMC6037685; doi:10.1038/s41598-018-28496-6)
Supplement: Supplementary file 1 — Supplementary Information [file 41598_2018_28496_MOESM1_ESM.pdf]

# Integrated Brain Atlas for Unbiased Mapping of Nervous System Effects Following Liraglutide Treatment

Casper Bo Jensen[1,2], Tess Tsai-Hsiu Lu[1], Sanaz Gabery[1], Kasper Marstal[2,4], Tomas Alanentalo[1], Aaron Jeffrey Mercer[3], Anda Cornea[3], Knut Conradsen[2], Jacob Hecksher-Soerensen[1], Anders Bjorholm Dahl[2], Lotte Bjerre Knudsen*[1], Anna Secher[1]

[1]Global Research, Novo Nordisk A/S, Måløv, Denmark;

[2]Image Analysis & Computer Graphics, Department of Applied Mathematics and Computer Science , Technical University of Denmark, Kgs. Lyngby, Denmark;

[3]Global Research, Novo Nordisk A/S, Seattle, USA;

[4]Biomedical Imaging Group Rotterdam (BIGR), Department of Radiology & Medical Informatics, Erasmus Medical Center, Rotterdam, Netherlands;

*Contact:

Lotte Bjerre Knudsen - lbkn@novonordisk.com

Novo Nordisk A/S, Novo Nordisk Park, DK-2760 Maaloev, Denmark.

## Supplementary Figures

# Integrated Brain Atlas for Unbiased Mapping of Nervous System Effects Following Liraglutide Treatment

- S1. **Image registration**
- S2. **Accuracy of image registration**
- S3. **Unmix signals**
- S4. **Average brain signals**
- S5. **Confocal images of liraglutide$^{Cy3}$**
- S6. **Enhanced ISH images**
- S7. **Liraglutide$^{VT750}$ in wild type vs *Glp-1r*$^{-/-}$ mice**
- S8. **c-Fos heat maps**
- S9. **Rank plot**

S1. **Image registration**

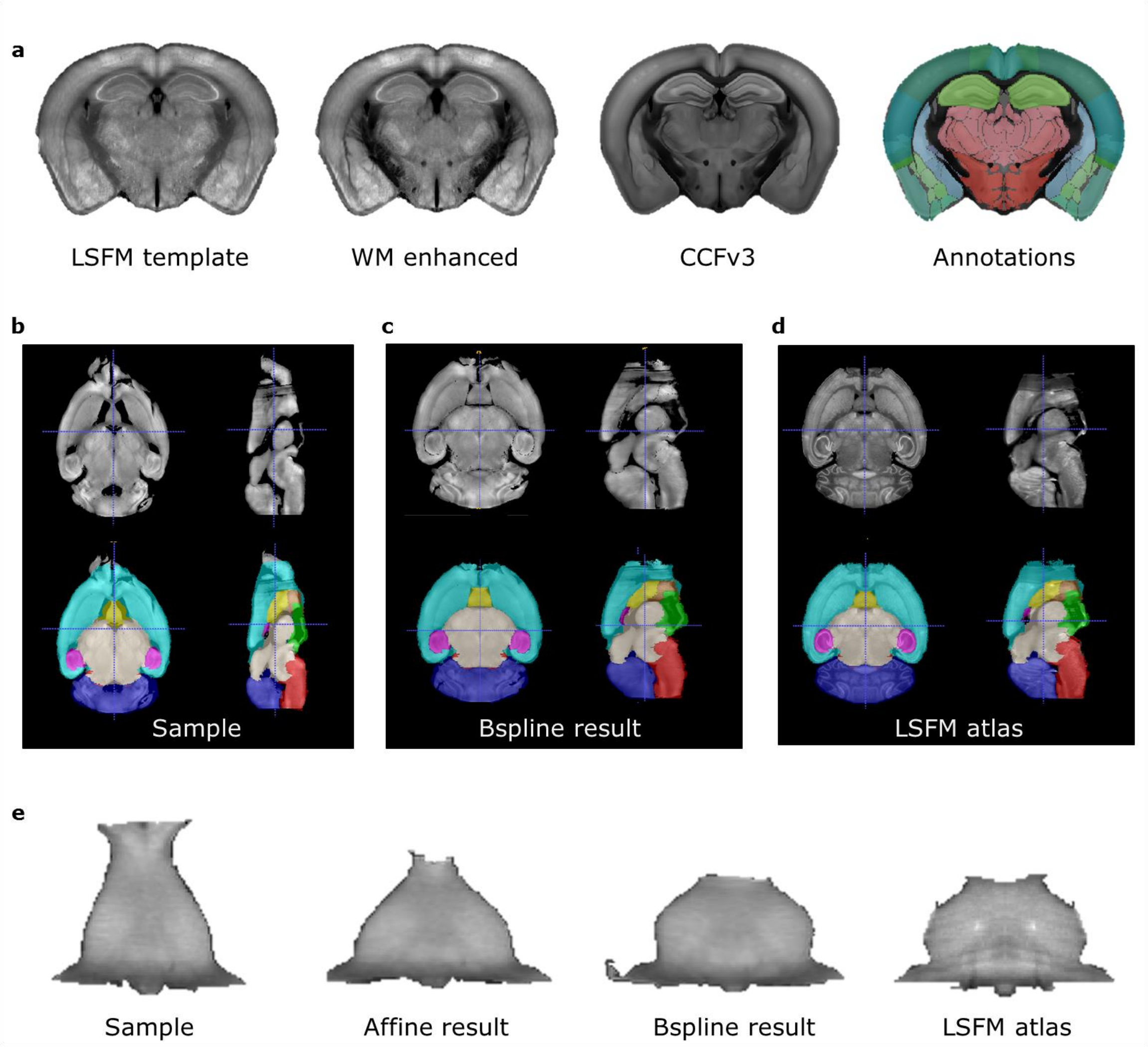


**Supplement Figure 1 I Image registration.** (**a**) A LSFM template was constructed from a representative C57Bl/6J male mouse by registration of the brain to the CCFv3 atlas[7] from AIBS. A white matter (WM) enhanced version of the LSFM template was constructed to increase the alignment quality. (**b**) Example of a brain sample to be registered to the LSFM template of the integrated brain atlas. Large deformations have occurred during the tissue processing prior to imaging. (**c**) The result of aligning the sample to the LSFM atlas. The registration was performed in separate parts to mitigate the large deformations. Separate affine and b-spline registration was performed for ten larger structures, here visualized by the overlay colours in the lower part of the figure. (**d**) LSFM atlas to which the brain sample was aligned. (**e**) Example of the registration performed for one of the larger structures in the alignment between the brain sample and LSFM atlas. Images are taken from the yellow structure shown in the lower part of (**b**)-(**d**).

S2. **Accuracy of image registration**

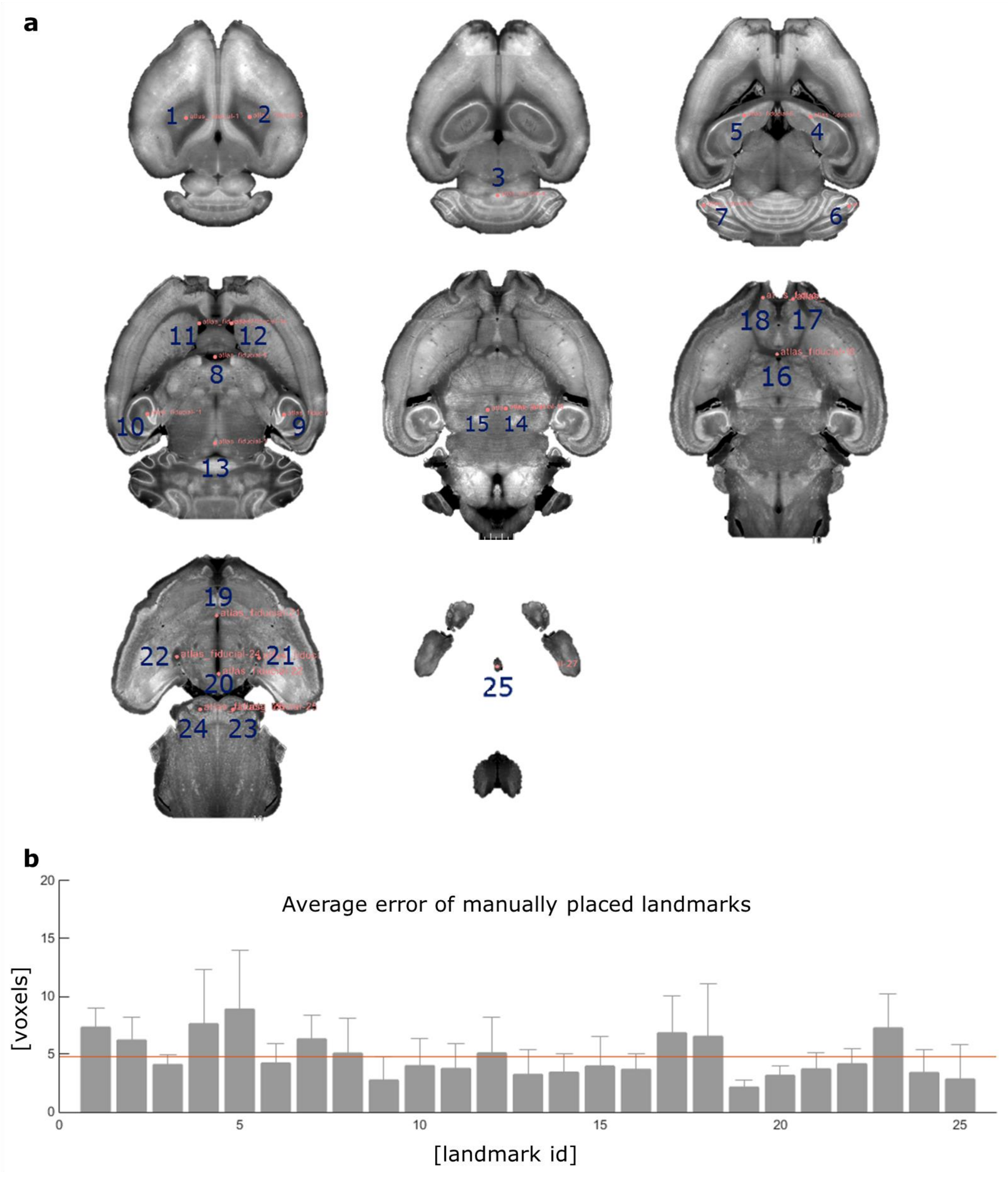


**Supplement Figure2 I Accuracy of image registration.** (**a**) The accuracy of the image registration for LSFM data was evaluated by manually placing 25 landmarks distributed throughout the LSFM template and comparing to corresponding landmarks placed in five brain samples after they had been registered to the LSFM template. Bar graph showing the mean error and standard deviation (n=5). The average error between the corresponding landmarks in samples and LSFM template was approximately 5 voxels corresponding to 100 µm.

S3. **Unmix signals**

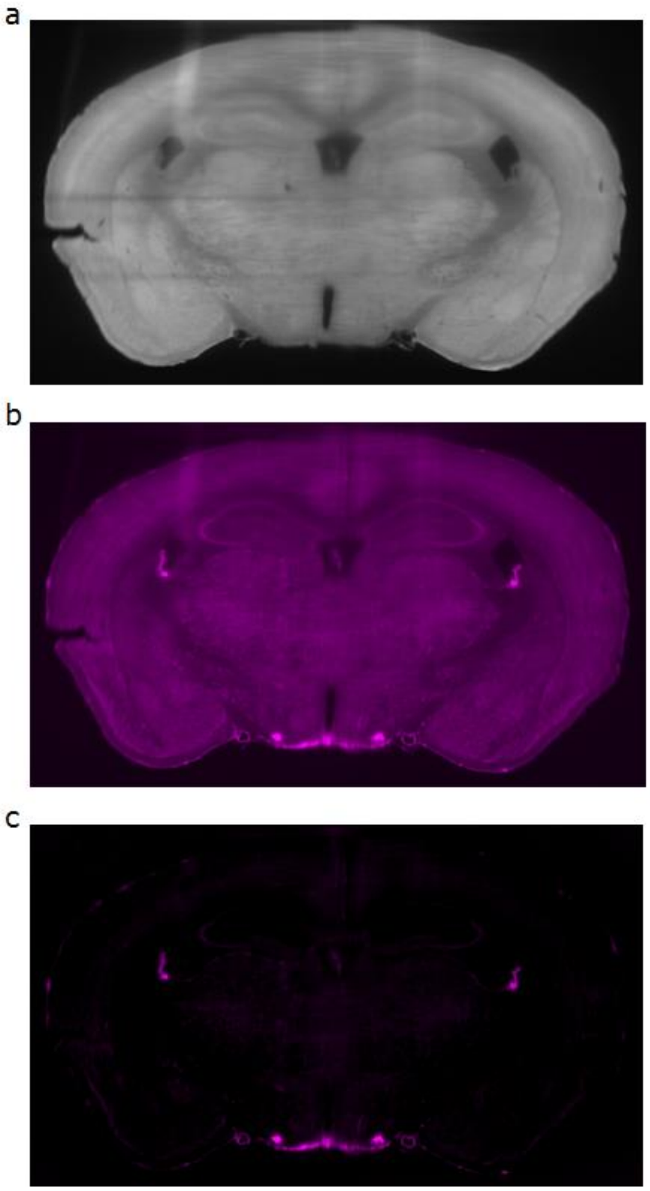


**Supplement Figure 3 I Unmix signals.** (**a**) Coronal projection of auto-fluorescence channel from liraglutide$^{VT750}$ dosed animal acquired with LSFM. (**b**) Coronal projection of specific channel from liraglutide$^{VT750}$ dosed animal. (**c**) Coronal projection of computed unmix channel from liraglutide$^{VT750}$ dosed animal.

S4. **Average brain signals**

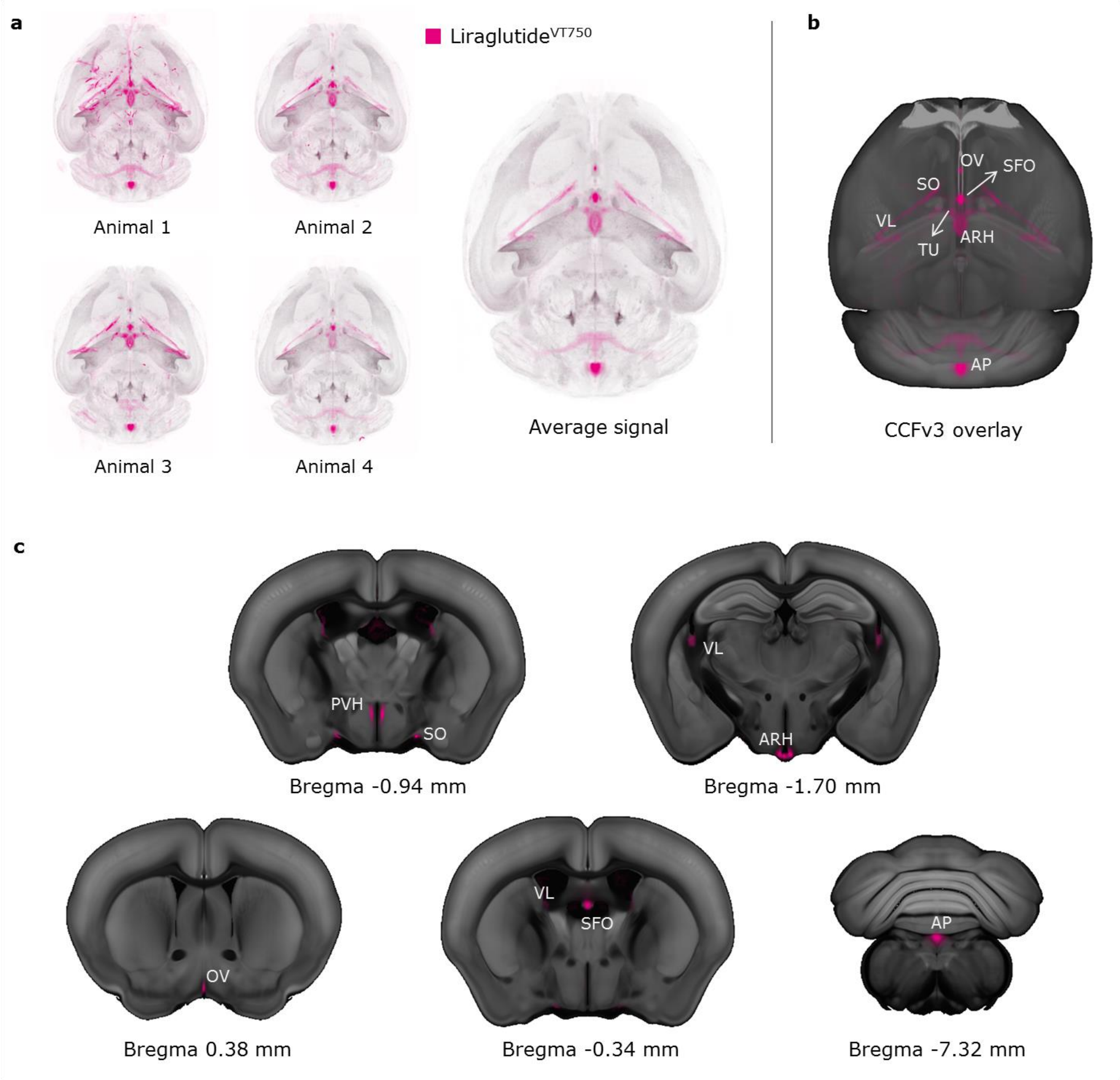


**Supplement Figure 4 I Average brain signals.** (**a**) Maximum intensity projection images of four individual brains from mice injected I.V. with liraglutide$^{VT750}$. The brains were registered to the atlas space and a voxel-wise average distribution signal representing the experiment group was computed. (**b**) The average signal from (a) overlaid onto the CCFv3 template[7]. (**c**) Coronal projection images constructed from (b). The color map was adjusted by a factor 2.5 in the top row to ease visualization of the lower signals in the non-circumventricular regions compared to the higher signals in the circumventricular regions (bottom row).

S5. **Confocal images of liraglutide$^{Cy3}$**

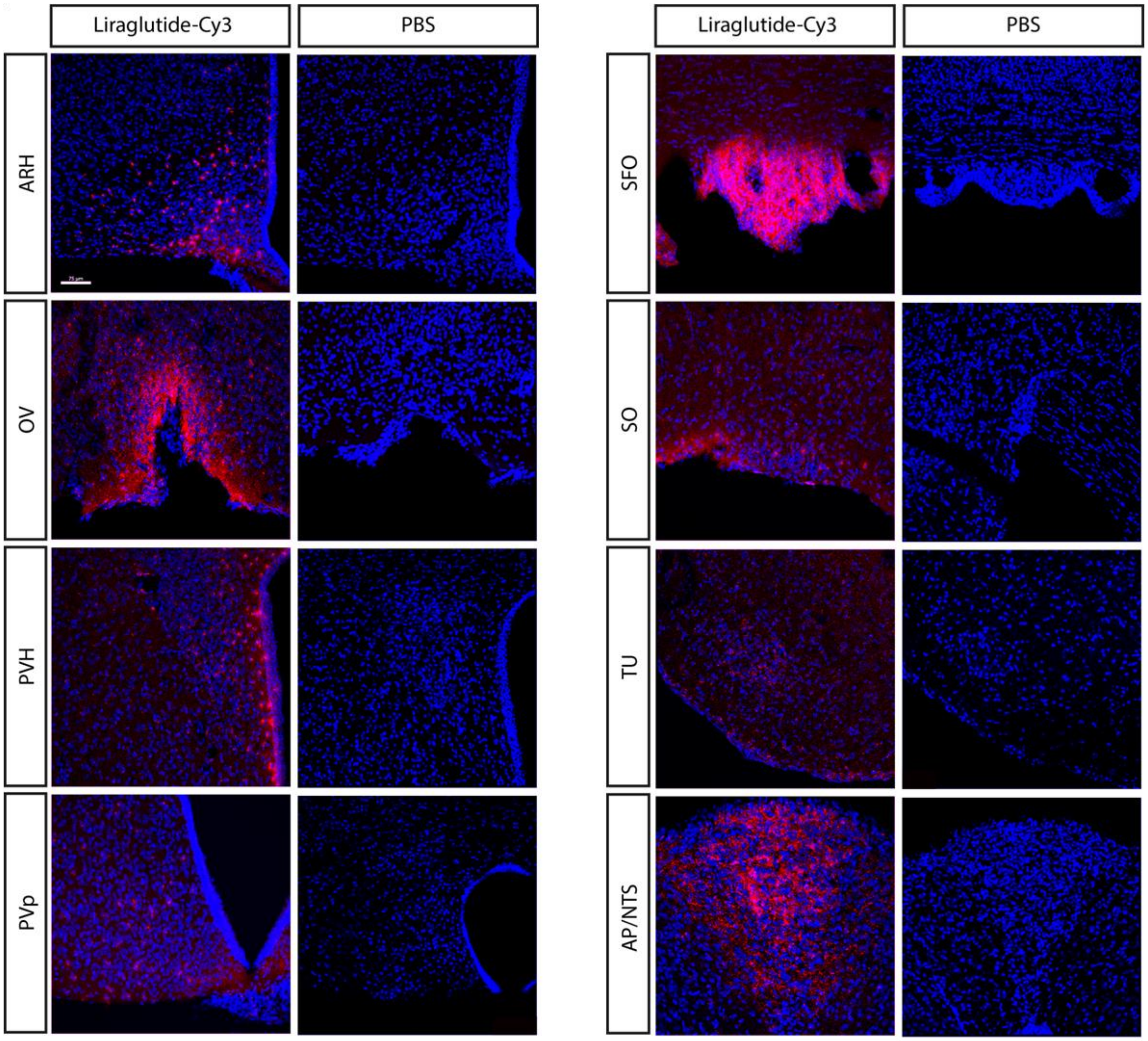


**Supplement Figure 5 I Confocal images of liraglutide$^{Cy3}$.** Overview of liraglutide$^{Cy3}$ in the mouse brain. High magnification confocal images from mice injected with liraglutide$^{Cy3}$. Brain regions where liraglutide$^{Cy3}$ (red) stained with DAPI nuclear stain (blue) were observed, compared to the corresponding region from an animal injected with vehicle (PBS).

S6. **Enhanced ISH images**

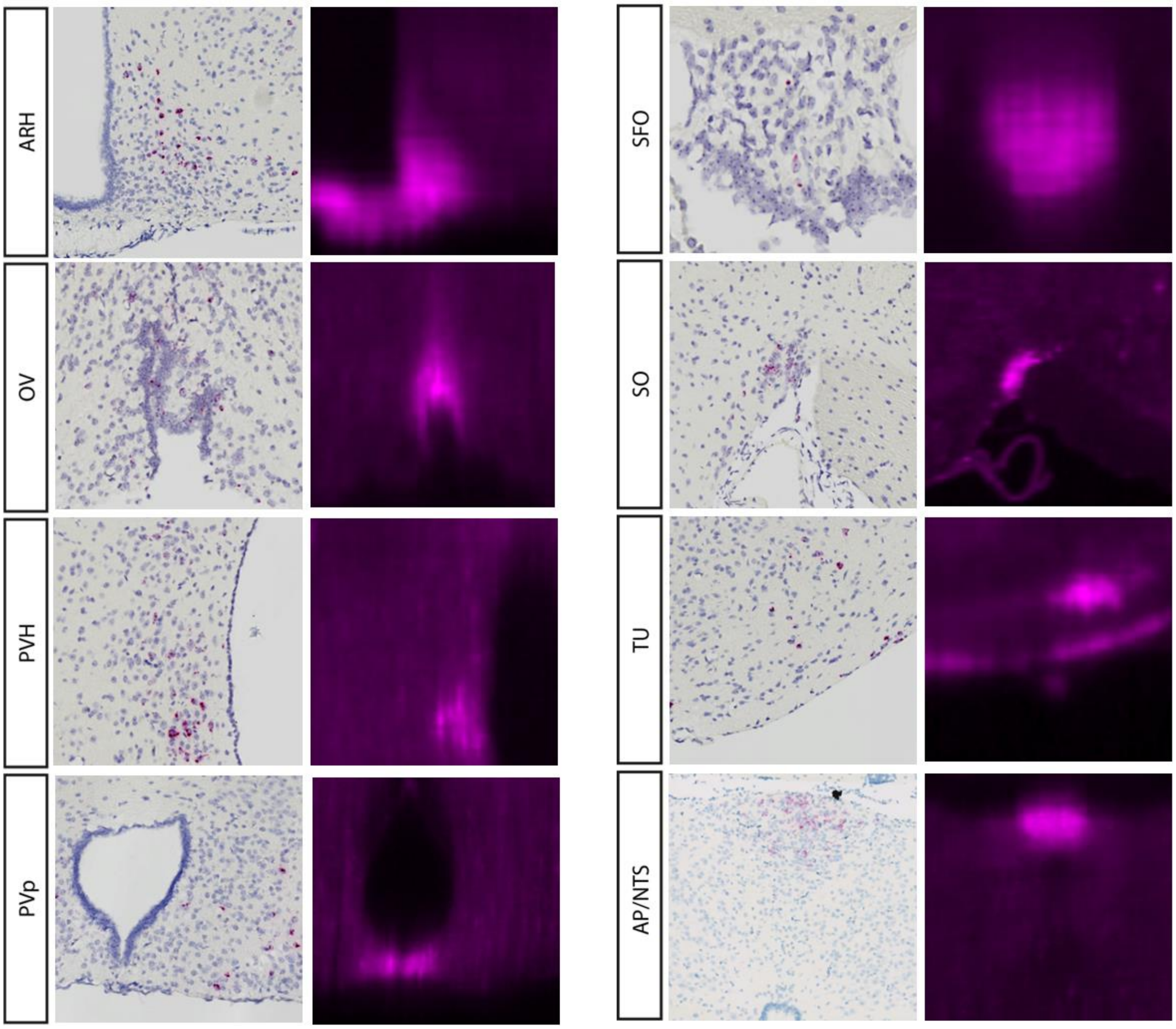


**Supplement Figure 6 I Enhanced ISH images.** Images show glp-1r expression in targeted brain regions. Overview of the glp-1r mRNA localization in a number of coronal sections throughout the C57/BL6 mouse brain. High magnification slide scanner images from untreated mice. Panel shows selected brain regions with glp-1r mRNA (red) counter stained with Mayer's hematoxylin (blue). The ISH images are compared to corresponding coronal projection images from a liraglutide$^{VT750}$ distribution signal acquired with LSFM (pink).

S7. **Liraglutide$^{VT750}$ in wild type vs *Glp-1r$^{-/-}$* mice**

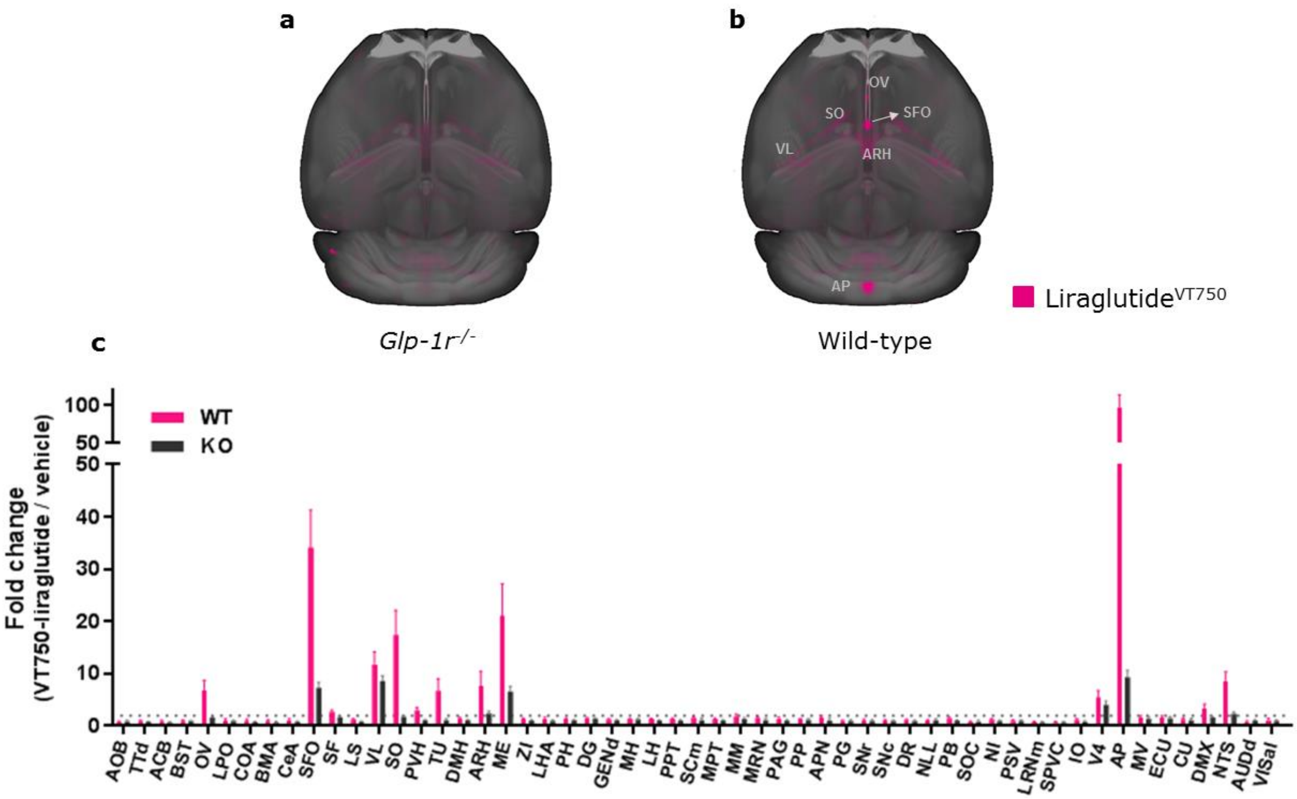


**Supplement Figure 7 I Liraglutide$^{VT750}$ in wild type vs *Glp-1r$^{-/-}$* mice.** (**a**) Maximum intensity projection of average liraglutide$^{VT750}$ signal following administration in *Glp-1r$^{-/-}$* animals overlaid onto the CCfv3 template[7]. (**b**) Maximum intensity projection of liraglutide$^{VT750}$ signal following administration in wild-type animals overlaid onto the CCfv3 template[7]. (**c**) Bar graph showing mean fold change and standard deviation of vehicle normalized signals (total fluorescence) from liraglutide$^{VT750}$ injected wild-type (WT) and *Glp-1r$^{-/-}$* (KO) animals.

S8. **c-Fos heat maps**

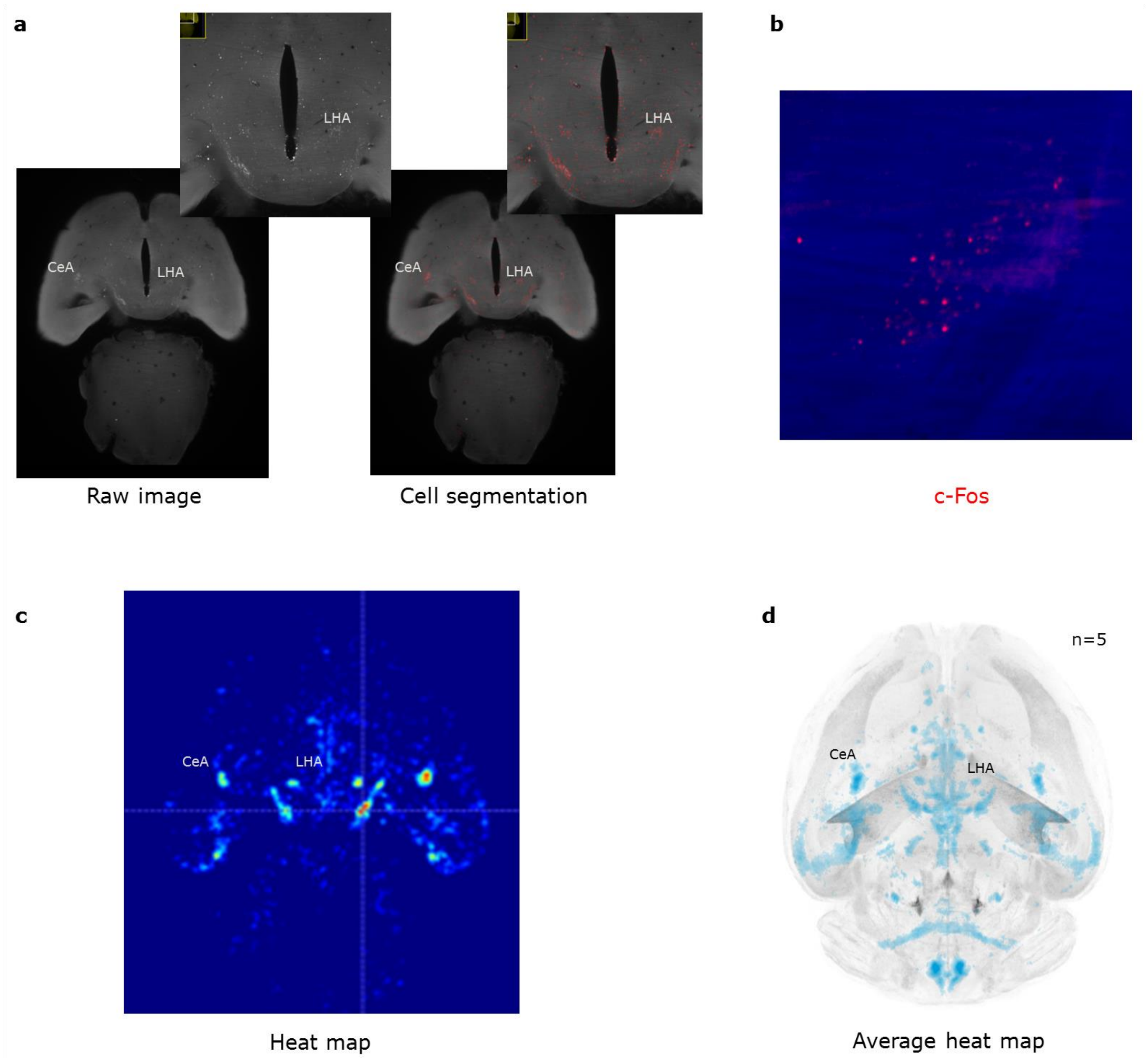


**Supplement Figure 8 I c-Fos heat maps.** (**a**) Raw LSFM image from a liraglutide injected mouse. The whole brain was stained for c-Fos prior to LSFM imaging. The red overlay shows the result of classifying cells positive for c-Fos. (**b**) High resolution image showing c-Fos positive cells (red). (**c**) c-Fos heat map constructed from the image seen in (a). Red color indicates high density of c-Fos positive cells. (**d**) Maximum intensity projection image of average c-Fos signal from five animals injected with liraglutide.

S9. **Rank plot**

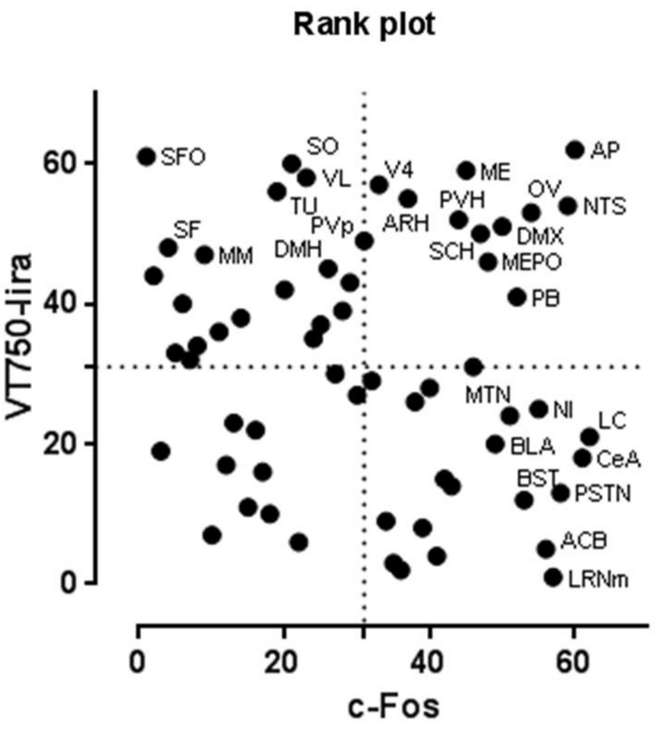


**Supplement Figure 9 I Rank plot.** Rank plot comparing c-Fos activation data from Fig. 4c with liraglutide$^{VT750}$ distribution data from Fig. 2b. The brain regions were ranked based on their fold change value and these rankings were used as x and y coordinates in the plot. Top right corner contains brain regions where a direct activation from liraglutide is most plausible.

## Supplementary Tables

# Integrated Brain Atlas for Unbiased Mapping of Nervous System Effects Following Liraglutide Treatment

- T1. **Brain region abbreviations**
- T2. **Most overlapping connectivity maps from AIBS**
- T3. **Multiple t-tests, brain distribution**
- T4. **Multiple t-tests, brain activation**

T1. **Brain region abbreviations**

| Abbreviation | Full name | Abbreviation | Full name |
|---|---|---|---|
| ACB | Nucleus accumbens | MM | Medial mammillary nucleus |
| AHN | Anterior hypothalamic nucleus | MPT | Medial pretectal area |
| AOB | Accessory olfactory bulb | MRN | Midbrain reticular nucleus |
| AP | Area postrema | MTN | Midline group of the dorsal thalamus |
| ARH | Arcuate hypothalamic nucleus | NI | Nucleus incertus |
| AUDd | Dorsal auditory area | NLL | Nucleus of the lateral lemniscus |
| BLA | Basolateral amygdalar nucleus | NTS | Nucleus of the solitary tract |
| BMA | Basomedial amygdalar nucleus | OV | Vascular organ of the lamina terminalis |
| BST | Bed nuclei of the stria terminalis | PAG | Periaqueductal gray |
| CA1 | Field CA1 | PB | Parabrachial nucleus |
| CA2 | Field CA2 | PCG | Pontine central gray |
| CA3 | Field CA3 | PG | Pontine gray |
| CEA | Central amygdalar nucleus | PH | Posterior hypothalamic nucleus |
| COA | Cortical amygdalar area | PSTN | Parasubthalamic nucleus |
| CU | Cuneate nucleus | PVH | Paraventricular hypothalamic nucleus |
| DG | Dentate gyrus | PVp | Periventricular hypothalamic nucleus, posterior part |
| DMH | Dorsomedial nucleus of the hypothalamus | PVT | Paraventricular nucleus of the thalamus |
| DMX | Dorsal motor nucleus of the vagus nerve | RE | Nucleus of reunions |
| DR | Dorsal nucleus raphe | RHP | Retrohippocampal region |
| ECT | Ectorhinal area | SCH | Suprachiasmatic nucleus |
| ECU | External cuneate nucleus | SCm | Superior colliculus, motor related |
| GENd | Geniculate group, dorsal thalamus | SF | Septofimbrial nucleus |
| GR | Gracile nucleus | SFO | Subfornical organ |
| IC | Inferior colliculus | SNc | Substantia nigra, compact part |
| LC | Locus ceruleus | SNr | Substantia nigra, reticular part |
| LH | Lateral habenula | SO | Supraoptic nucleus |
| LHA | Lateral hypothalamic area | TTd | Taenia tecta, dorsal part |
| LPO | Lateral preoptic area | TU | Tuberal nucleus |
| LRNm | Lateral reticular nucleus, magnocellular part | V4 | fourth ventricle |
| LS | Lateral septal nucleus | VL | lateral ventricle |
| ME | Median eminence | VMH | Ventromedial hypothalamic nucleus |
| MEA | Medial amygdalar nucleus | VTA | Ventral tegmental area |
| MEPO | Median preoptic nucleus | ZI | Zona incerta |
| MH | Medial habenula | | |

**Supplementary Table 1 I Brain region abbreviations.** Full name of brain region abbreviations used in this manuscript. Brain region names follow the nomenclature of the AIBS CCFv3 atlas[7].

T2. **Most overlapping connectivity maps from AIBS**

| Rank | Structure | Mouse line | Experiment id |
|---|---|---|---|
| 1 | NTS/DMX | Rasgrf2-T2A-dCre | 310853453 |
| 2 | CEA | C57BL/6J | 112459547 |
| 3 | PB | Calb2-IRES-Cre | 183284388 |
| 4 | PB | Slc17a6-IRES-Cre | 305404551 |
| 5 | GR/NTS | Gal-Cre_KI87 | 175816975 |
| 6 | PB | Npr3-IRES2-Cre | 545427588 |
| 7 | PAG | Gad2-IRES-Cre | 302053755 |
| 8 | PB | Chat-IRES-Cre-neo | 264565965 |
| 9 | GR/NTS | C57BL/6J | 159648854 |
| 10 | AHN | Slc18a2-Cre_OZ14 | 301673462 |
| 11 | PVT | Ntrk1-IRES-Cre | 263106751 |
| 12 | PB | Chat-IRES-Cre-neo | 183901489 |
| 13 | NTS/DMX | Pnmt-Cre | 155735108 |
| 14 | VMH/TU | C57BL/6J | 112228391 |
| 15 | CEA | Prkcd-GluCla-CFP-IRES-Cre | 265945645 |
| 16 | PVH | Tac1-IRES2-Cre | 300888673 |
| 17 | MEA | C57BL/6J | 157550122 |
| 18 | BST | Ppp1r17-Cre_NL146 | 267764292 |
| 19 | NTS/DMX | Ppp1r17-Cre_NL146 | 268323342 |
| 20 | NTS/DMX | Tac1-IRES2-Cre | 180525136 |
| 21 | DMH | Sim1-Cre | 159222295 |
| 22 | PVT | C57BL/6J | 120875111 |
| 23 | VMH/LHA | Erbb4-T2A-CreERT2 | 277854916 |
| 24 | MEA | Adcyap1-2A-Cre | 272819994 |
| 25 | BLA/MEA | Crh-IRES-Cre_BL | 277856332 |
| 26 | AHN/LHA | Nos1-CreERT2 | 304998039 |
| 27 | PVT | Grm2-Cre_MR90 | 183225830 |
| 28 | CEA/MEA | Crh-IRES-Cre_ZJH | 267152406 |
| 29 | ARH | Pomc-Cre_BL | 263369222 |
| 30 | RE/PVH | Rasgrf2-T2A-dCre | 310976160 |

**Supplementary Table 2 I Most overlapping connectivity maps from AIBS.** (**a**) List of the 30 most overlapping connectivity maps from AIBS compared to the liraglutide specific c-Fos increase following acute administration. The "Structure" column indicates the primary injection site where the virus used to trace the connections were injected. The "Mouse line" column indicates the mouse line used for the specific tracing experiment. The "Experiment id" column shows a unique AIBS id number for each experiment. All connectivity maps[6] were downloaded from the AIBS data portal.

T3. **Multiple t-tests, brain distribution**

| Structure | Discovery? | P value | q value |
|---|---|---|---|
| AOB | No | 0.298288 | 0.513718 |
| AUDd | No | 0.457365 | 0.659456 |
| BLA | No | 0.799363 | 0.875941 |
| BMA | No | 0.987891 | 0.987891 |
| CA1 | No | 0.787127 | 0.875941 |
| CA2 | No | 0.587313 | 0.74313 |
| CA3 | No | 0.497779 | 0.679617 |
| COA | No | 0.778259 | 0.875941 |
| DG | No | 0.030414 | 0.099245 |
| ECT | No | 0.408925 | 0.618375 |
| RHP | No | 0.603671 | 0.748553 |
| TTd | No | 0.524749 | 0.690585 |
| ACB | No | 0.504232 | 0.679617 |
| BST | No | 0.855094 | 0.902401 |
| CeA | No | 0.701597 | 0.838174 |
| LS | No | 0.39843 | 0.617567 |
| SF | Yes | 0.004443 | 0.030606 |
| ARH | Yes | 0.009872 | 0.040806 |
| DMH | Yes | 0.0028 | 0.02893 |
| LHA | No | 0.080797 | 0.2178 |
| LPO | No | 0.494588 | 0.679617 |
| ME | Yes | 0.008816 | 0.040806 |
| MEPO | No | 0.099089 | 0.231437 |
| MM | No | 0.112884 | 0.236069 |
| OV | Yes | 0.005924 | 0.036731 |
| PH | No | 0.101531 | 0.231437 |
| PSTN | No | 0.858737 | 0.902401 |
| PVH | Yes | 0.001346 | 0.021867 |
| PVp | Yes | 0.007777 | 0.040184 |
| SCH | No | 0.265946 | 0.499657 |
| SFO | Yes | 0.00166 | 0.021867 |
| SO | Yes | 0.003336 | 0.029549 |
| TU | Yes | 0.009586 | 0.040806 |
| VMH | No | 0.114227 | 0.236069 |
| ZI | No | 0.097973 | 0.231437 |
| GENd | No | 0.015074 | 0.058412 |
| LH | No | 0.092259 | 0.231437 |
| MH | No | 0.019678 | 0.071766 |
| MTN | No | 0.160826 | 0.3116 |
| DR | No | 0.286786 | 0.508021 |

| | | | |
|---|---|---|---|
| IC | No | 0.274757 | 0.501027 |
| MPT | No | 0.129791 | 0.259582 |
| MRN | No | 0.10452 | 0.231437 |
| PAG | No | 0.064576 | 0.200184 |
| SCm | No | 0.079241 | 0.2178 |
| SNc | No | 0.874257 | 0.903398 |
| SNr | No | 0.716504 | 0.838174 |
| VTA | No | 0.317783 | 0.518488 |
| LC | No | 0.534646 | 0.690585 |
| NI | No | 0.31211 | 0.518488 |
| NLL | No | 0.805301 | 0.875941 |
| PB | No | 0.023846 | 0.082135 |
| PCG | No | 0.076102 | 0.2178 |
| PG | No | 0.964062 | 0.979866 |
| AP | Yes | 0.000099 | 0.006119 |
| CU | No | 0.33423 | 0.53134 |
| DMX | Yes | 0.00724 | 0.040184 |
| ECU | No | 0.708547 | 0.838174 |
| LRNm | No | 0.420513 | 0.620758 |
| NTS | Yes | 0.001763 | 0.021867 |
| VL | Yes | 0.000333 | 0.010334 |
| V4 | Yes | 0.00411 | 0.030606 |

**Supplementary Table 3 I Multiple t-tests, brain distribution.** Statistical results of multiple t-tests on all brain regions from Figure 2b. Discovery was determined using the Original FDR method of Benjamini and Hochberg, with Q = 5%.Each row was analyzed individually, without assuming a consistent SD.

T4. **Multiple t-tests, brain activation**

| Structure | Discovery? | P value | q value |
|---|---|---|---|
| AOB | No | 0.649471 | 0.774369 |
| AUDd | No | 0.401123 | 0.604277 |
| BLA | Yes | 0.014399 | 0.117397 |
| BMA | No | 0.548283 | 0.686395 |
| CA1 | No | 0.094397 | 0.36579 |
| CA2 | No | 0.320452 | 0.584353 |
| CA3 | No | 0.3919 | 0.604277 |
| COA | No | 0.849841 | 0.893053 |
| DG | No | 0.288082 | 0.541245 |
| ECT | No | 0.273741 | 0.53315 |
| RHP | No | 0.173924 | 0.448171 |
| TTd | No | 0.525741 | 0.686395 |
| ACB | No | 0.126403 | 0.385527 |
| BST | Yes | 0.013678 | 0.117397 |
| CeA | Yes | 0.0001 | 0.006214 |
| LS | No | 0.398816 | 0.604277 |
| SF | No | 0.200579 | 0.448171 |
| ARH | Yes | 0.028769 | 0.178601 |
| DMH | No | 0.372062 | 0.604277 |
| LHA | No | 0.235671 | 0.503848 |
| LPO | No | 0.13118 | 0.385527 |
| ME | No | 0.044773 | 0.252355 |
| MEPO | No | 0.1368 | 0.385527 |
| MM | No | 0.836736 | 0.893053 |
| OV | No | 0.094026 | 0.36579 |
| PH | No | 0.3795 | 0.604277 |
| PSTN | Yes | 0.003535 | 0.054791 |
| PVH | No | 0.124041 | 0.385527 |
| PVp | No | 0.102325 | 0.373186 |
| SCH | No | 0.083844 | 0.36579 |
| SFO | No | 0.275174 | 0.53315 |
| SO | No | 0.553544 | 0.686395 |
| TU | No | 0.474333 | 0.66812 |
| VMH | No | 0.393079 | 0.604277 |
| ZI | No | 0.519105 | 0.686395 |
| GENd | No | 0.976057 | 0.976057 |
| LH | No | 0.951222 | 0.966816 |
| MH | No | 0.81227 | 0.891098 |
| MTN | Yes | 0.015148 | 0.117397 |
| DR | No | 0.804906 | 0.891098 |

| | | | |
|---|---|---|---|
| IC | No | 0.484926 | 0.66812 |
| MPT | No | 0.819235 | 0.891098 |
| MRN | No | 0.536862 | 0.686395 |
| PAG | No | 0.873871 | 0.903 |
| SCm | No | 0.182647 | 0.448171 |
| SNc | No | 0.08842 | 0.36579 |
| SNr | No | 0.805588 | 0.891098 |
| VTA | No | 0.2024 | 0.448171 |
| LC | Yes | 0.001961 | 0.040529 |
| NI | No | 0.118438 | 0.385527 |
| NLL | No | 0.270209 | 0.53315 |
| PB | Yes | 0.028807 | 0.178601 |
| PCG | No | 0.158017 | 0.42596 |
| PG | No | 0.447606 | 0.645386 |
| AP | Yes | 0.008367 | 0.103745 |
| CU | No | 0.568752 | 0.691424 |
| DMX | No | 0.195675 | 0.448171 |
| ECU | No | 0.718 | 0.839924 |
| LRNm | No | 0.071984 | 0.36579 |
| NTS | Yes | 0.000793 | 0.024595 |
| VL | No | 0.409349 | 0.604277 |
| V4 | No | 0.402187 | 0.604277 |

**Supplementary Table 4 I  Multiple t-tests, brain activation.** Statistical results of multiple t-tests on all brain regions from Figure 5c. Discovery was determined using the Original FDR method of Benjamini and Hochberg, with Q = 20%.Each row was analyzed individually, without assuming a consistent SD.

## Supplementary Movies

# Integrated Brain Atlas for Unbiased Mapping of Nervous System Effects Following Liraglutide Treatment

- M1. **Comparing liraglutide$^{VT750}$ brain distribution with liraglutide specific c-Fos increase**
- M2. **Comparing liraglutide specific c-Fos increase with AIBS connectivity maps**

M1. **Comparing liraglutide$^{VT750}$ brain distribution with liraglutide specific c-Fos increase**

Average brain distribution of liraglutide$^{VT750}$ (pink) overlaid with liraglutide specific c-Fos increase (blue). Signals were imported into the integrated brain atlas for visualization.

M2. **Comparing liraglutide specific c-Fos increase with AIBS connectivity maps**

Liraglutide specific c-Fos increase (blue) overlaid with glutamatergic projections from the parabrachial nucleus (yellow), and PKD-delta+ projections from the central amygdalar nucleus (red). Signals were imported into the integrated brain atlas for visualization.
